# Genomic Hotspots for Adaptation: The Population Genetics of Müllerian Mimicry in the *Heliconius melpomene* Clade

**DOI:** 10.1371/journal.pgen.1000794

**Published:** 2010-02-05

**Authors:** Simon W. Baxter, Nicola J. Nadeau, Luana S. Maroja, Paul Wilkinson, Brian A. Counterman, Anna Dawson, Margarita Beltran, Silvia Perez-Espona, Nicola Chamberlain, Laura Ferguson, Richard Clark, Claire Davidson, Rebecca Glithero, James Mallet, W. Owen McMillan, Marcus Kronforst, Mathieu Joron, Richard H. ffrench-Constant, Chris D. Jiggins

**Affiliations:** 1 Department of Zoology, University of Cambridge, Cambridge, United Kingdom; 2 Smithsonian Tropical Research Institute, Balboa, Panama; 3 School of Biosciences, University of Exeter in Cornwall, Penryn, United Kingdom; 4 Department of Genetics, North Carolina State University, Raleigh, North Carolina, United States of America; 5 School of Biological Sciences, University of Bristol, Bristol, United Kingdom; 6 Harvard FAS Center for Systems Biology, Harvard University, Cambridge, Massachusetts, United States of America; 7 The Wellcome Trust Sanger Institute, Cambridge, United Kingdom; 8 The Galton Laboratory, University College London, London, United Kingdom; 9 Département Systématique et Evolution, Muséum National d'Histoire Naturelle, Paris, France; University of Arizona, United States of America

## Abstract

Wing patterning in *Heliconius* butterflies is a longstanding example of both Müllerian mimicry and phenotypic radiation under strong natural selection. The loci controlling such patterns are “hotspots” for adaptive evolution with great allelic diversity across different species in the genus. We characterise nucleotide variation, genotype-by-phenotype associations, linkage disequilibrium, and candidate gene expression at two loci and across multiple hybrid zones in *Heliconius melpomene* and relatives. Alleles at *HmB* control the presence or absence of the red forewing band, while alleles at *HmYb* control the yellow hindwing bar. Across *HmYb* two regions, separated by ∼100 kb, show significant genotype-by-phenotype associations that are replicated across independent hybrid zones. In contrast, at *HmB* a single peak of association indicates the likely position of functional sites at three genes, encoding a kinesin, a G-protein coupled receptor, and an mRNA splicing factor. At both *HmYb* and *HmB* there is evidence for enhanced linkage disequilibrium (LD) between associated sites separated by up to 14 kb, suggesting that multiple sites are under selection. However, there was no evidence for reduced variation or deviations from neutrality that might indicate a recent selective sweep, consistent with these alleles being relatively old. Of the three genes showing an association with the *HmB* locus, the *kinesin* shows differences in wing disc expression between races that are replicated in the co-mimic, *Heliconius erato*, providing striking evidence for parallel changes in gene expression between Müllerian co-mimics. Wing patterning loci in *Heliconius melpomene* therefore show a haplotype structure maintained by selection, but no evidence for a recent selective sweep. The complex genetic pattern contrasts with the simple genetic basis of many adaptive traits studied previously, but may provide a better model for most adaptation in natural populations that has arisen over millions rather than tens of years.

## Author Summary

The diversity of wing patterns in *Heliconius* butterflies is a longstanding example of both Müllerian mimicry and adaptive radiation. The genetic regions controlling such patterns are “hotspots” for adaptive evolution, with small regions of the genome controlling major changes in wing pattern. Across multiple hybrid zones in *Heliconius melpomene* and related species, we no find no strong population signal of recent selection. Nonetheless, we find significant associations between genetic variation and wing pattern at multiple sites. This suggests patterning alleles are relatively old, and might be a better model for most natural adaptation, in contrast to the simple genetic basis of recent human-induced selection such as pesticide resistance. Strikingly, across the region controlling the red forewing band, a very strong association with phenotype implicates three genes as potentially being involved in control of wing pattern. One of these, a kinesin gene, shows parallel differences in expression levels between divergent forms in the two mimetic species, making it a strong candidate for control of wing pattern. These results show that mimicry involves parallel changes in gene expression and strongly suggest a role for this gene in control of wing pattern.

## Introduction

One of the central outstanding questions in evolutionary biology concerns the predictability of evolution. Specifically, can we predict the number and effect size of genes involved in evolution, and the types of genetic changes that underlie particular kinds of evolution? Striking empirical examples of repeated use of the same genes in similar evolutionary changes suggest that common patterns may emerge even across distantly related taxa [1],[2]. A few such systems are well understood, for example the genetic changes involved in melanic pigmentation in vertebrates such as rock pocket and beach mice [3],[4], or the adaptation of stickleback fish to freshwater habitats involving loss of spines and lateral plates [5],[6]. Some general patterns are beginning to emerge from such studies. First, a few loci of major phenotypic effect are commonly involved in adaptation, and second, the same genes can be involved repeatedly across divergent lineages [2]. In addition, both cis-regulatory, structural and null mutations can play a role in adaptation, although intriguingly there is a suggestion that cis-regulatory change may be more important in inter-specific versus intra-specific adaptation [7]. To date, however, these patterns are inferred from just a handful of well-studied examples.

*Heliconius* butterflies offer an excellent model system in which to address these questions as their wing patterns are under simple Mendelian genetic control and subject to strong natural selection [8]. The bright colour patterns function to advertise the toxicity of the butterflies to predators leading to stabilising selection and Müllerian mimicry, whereby sympatric species evolve a common colour pattern in order to share the cost of predator learning [9]. Strikingly, wing patterns are controlled by a small number of genomic ‘hotspots’ with a disproportionate influence on both divergent and convergent wing patterns [8],[10]. Classic crossing experiments have established that tightly linked loci on two linkage groups control most of the variation both within *Heliconius melpomene* and between closely related species [8]. More recently, the development of molecular markers has facilitated comparative mapping between more distantly related species. This has shown homology in genomic location of patterning loci between both the co-mimics *H. melpomene* and *Heliconius erato*, and a third species, *Heliconius numata*, with entirely different wing patterns [11]. Thus, much as the MC1R locus is repeatedly involved in adaptive melanic pigmentation in vertebrates [2], repeated involvement of the same genomic regions also underlies the complex wing patterns of *Heliconius* butterflies. However, the specific genetic changes underlying phenotypic diversity in *Heliconius* remain unknown.

Here and in a companion paper [12] we take advantage of *Heliconius* mimicry as a system to study the population genetic patterns around loci involved in parallel phenotypic changes. Patterns of genetic variation in natural populations are commonly used to identify genes under selection, either through association studies that correlate genetic and phenotypic variation [13], or through genome-wide scans for reduced variation or other signals of recent selection [14]–[16]. Such studies rely on the fact that selection on a locus also influences patterns of genetic variation at surrounding loci, through genetic ‘hitchhiking’ [17]. For example, after the recent and rapid rise of advantageous alleles, such during the evolution of insecticide resistance, there is often very strong linkage disequilibrium and dramatic reductions in nucleotide variability [18],[19]. In contrast, morphological adaptation in the wild is likely to be more ancient, with selection sustained over longer time periods, such that the influence on surrounding genetic variation may be more difficult to detect. Thus, between morphologically divergent forms the classic signature of a selective sweep may have decayed, due to the combined effects of recombination and accumulation of derived mutations.

In *Heliconius*, however, we can take advantage of natural hybrid zones, where admixture occurs between divergent geographic races, to carry out analysis of genotype-by-phenotype association. In particular, studies of a parallel hybrid zone in Peru between races of the co-mimics *H. erato* and *H. melpomene* have estimated that selection is extremely strong, on the order of a 20–50% reduced survival of foreign colour pattern morphs [20]. Crossing experiments have shown that *Heliconius* races typically differ at just two or three loci with major phenotypic effect on wing pattern [21]. Theory predicts that in such zones where relatively few genes are under strong selection, the barrier to gene flow at the rest of the genome will be weak [22]. This contrasts with many hybrid zones where many genes across the genome are under selection, leading to strong linkage disequilibrium and a genome-wide barrier to gene flow [23]–[25]. Thus, the situation in *Heliconius* offers an unusual opportunity to study populations that are strongly morphologically differentiated but nonetheless free to exchange genes across most of the genome. Previous demographic studies of *Heliconius* have demonstrated genetic differentiation on a regional scale, for example between the Guiana Shield, Amazon basin and Pacific slopes, but extensive gene flow between local populations ([26]–[29]; but see [30]). Thus, when comparing populations fixed for alternate wing patterns on either side of a narrow hybrid zone, we would predict little or no genetic differentiation across most of the genome. In contrast, around functional sites controlling wing patterning we expect fixed differences between pattern races, offering a powerful system for detecting sites associated with adaptation even in the absence of the signal of a classic ‘selective sweep’.

Specifically, we here take advantage of our recent positional cloning of two such loci located on different chromosomes, the *H. melpomene HmYb* and *HmB* loci. Alleles at these loci are responsible for controlling presence/absence of the hindwing yellow bar and red forewing band respectively. In this study, we fully sequence and annotate the *HmB* genomic region [31], and study patterns of genetic variation at both loci between species and races in the *H. melpomene* clade. Our work in *H. melpomene* has led to cloning of the corresponding region in *H. erato*, allowing for comparative analysis of both regions across species [12]. At both loci there is greater genetic divergence at markers linked to wing pattern as compared to unlinked regions, with significant peaks of genotype-by-phenotype association. Nonetheless, there is no strong signature of a recent selective sweep and sites associated with phenotype are interspersed with sites showing no such association, implying that wing patterning alleles are relatively ancient. However, a strong signature of divergence, and evidence for long-range haplotype structure associated with wing pattern divergence demonstrates a clear influence of selection on population variation. One of the three genes implicated at the *HmB* locus, a kinesin, shows striking differences in wing pattern expression between races and throughout wing development, making it a strong candidate for the *HmB* gene.

## Results

### BAC tile path across the *HmB* locus

**Figure 1:**
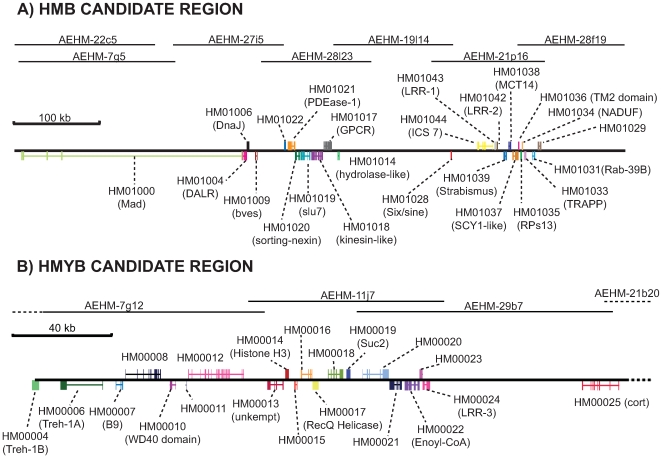
Annotation of the *HmB* and *HmYb* gene regions. Sequenced *H. melpomene* BAC clones across the 721 kb *HmB* locus and the 330 kb *HmYb* locus as defined by recombination mapping. In total 24 predicted genes were identified in the *HmB* region and 20 in the *HmYb* region. AEHM-22C5 and AEHM-7G5 are overlapping clones from different fingerprinted contigs, and both contain part of the *Mad* gene. Also see Table S1 for *HmB* annotation and ref. 65 for *HmYb* annotation.

We characterised the *HmB* locus by sequencing of 980 kb of *H. melpomene* genomic sequence from seven BAC clones, representing a 721 kb tile path, based on previous fine-mapping of this region [31]. All seven BAC clones were annotated by comparison with transcriptome sequence and protein databases, using a combination of manual BLAST comparison, the KAIKOGAAS lepidopteran genome annotation tool and the MAKER annotation pipeline [32]. Twenty-four genes with homology to known or predicted genes (BLAST cut off expect value 4.0e-08) were identified across the region, including several wing patterning candidates involved in development. Putative biological functions based on gene homology or predicted functional domains include cellular differentiation (*Mad*), protein binding (similar to *CG7872*, *GPCR*, *INCS7*), splicing (*slu7*), protein-protein interactions (leucine rich repeats or LRR), transport (*Monocarboxylate transporter 14*, *kinesin*), basic cell function (*RpS13*, *NADH*, *TPP5*) and a transcription factor (*six/sine*) (Figure 1 and Table S1). In addition, seven predicted transcription units, without introns or homology to known genes, were also identified. This complements our recent description of the *HmYb* genomic region, which has been narrowed down to a 323 kb genomic region containing 20 genes (Figure 1; [33]). Overall gene content and order is largely conserved across both regions between the co-mimic species, *H. erato* and *H. melpomene* (Figure S1). A direct comparison of gene order was not possible, as several *H. erato* BAC clones are not yet fully assembled, however a single copy of all 24 predicted *H. melpomene HmB* genes was identified in *H. erato* (data not shown), and a similar pattern is seen at *HmYb* where sequence is available for *H. erato*.

### Population structure between species and races

**Figure 2:**
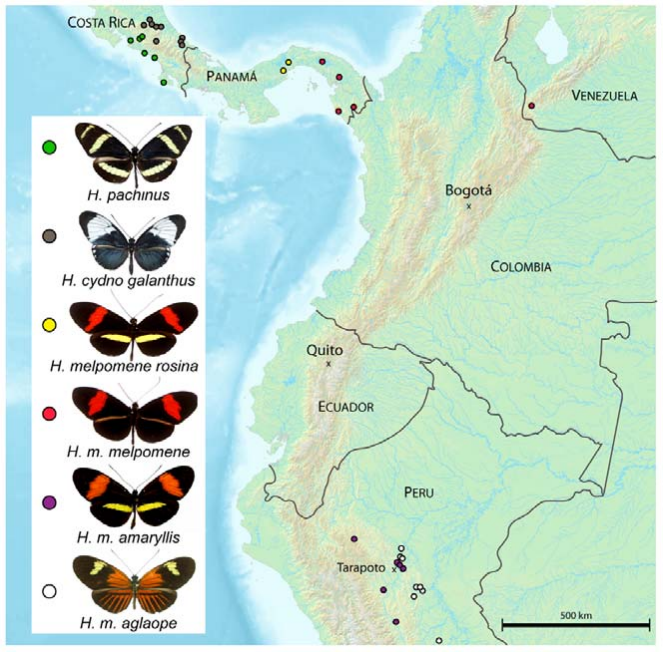
Locations of field collected samples used for population genetic analysis. *H.m. rosina*, *H.m. melpomene*, and *H.m. amaryllis* display the medial red forewing band controlled by a dominant allele at *HmB*. In addition, *H. pachinus*, *H.m. rosina*, and *H.m. amaryllis* display the yellow hindwing bar controlled by a recessive allele at *HmYb*.

**Figure 3:**
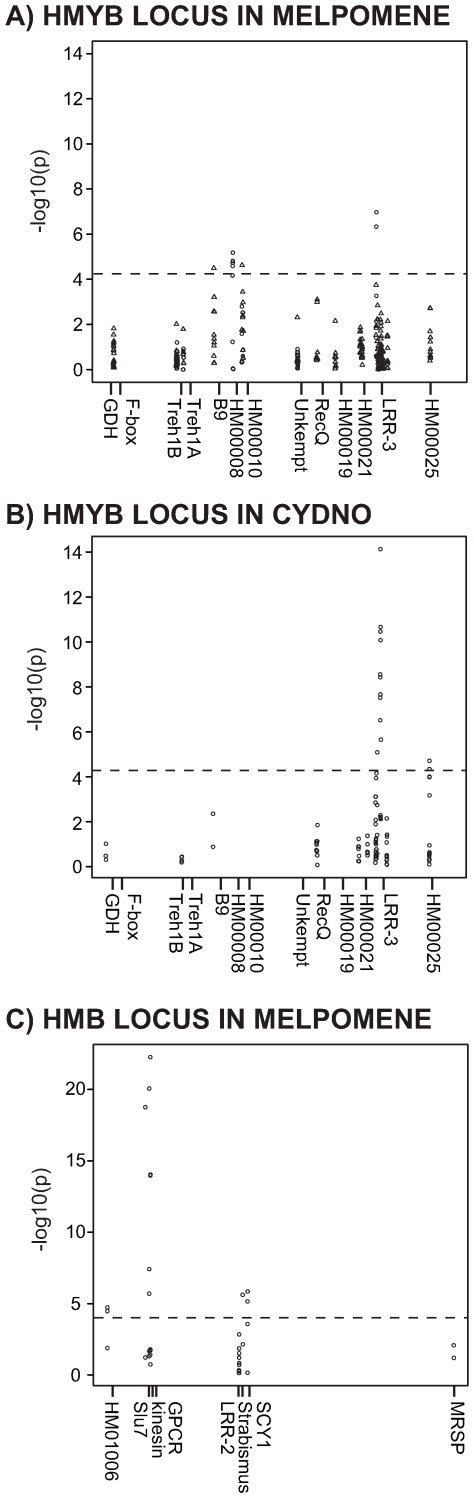
Genotype-by-phenotype associations across three phenotypic comparisons. (A) The *HmYb* region between *H. m. melpomene* and *H. m. rosina* (triangles) and between *H. m. aglaope* and *H. m. amaryllis* (circles), (B) The *HmYb* region between *H. pachinus* and *H. cydno*, and (C) the *HmB* region between *H. m. aglaope* and *H. m. amaryllis*. Significance cut-off was calculated using the Bonferroni correction, applied across all 866 SNPs tested.

We estimated population structure at *HmYb* markers for three pairwise population comparisons, two involving races of *H. melpomene* (*amaryllis* vs *aglaope* and *melpomene* vs *rosina*) and one pair of hybridizing species (*H. pachinus* vs. *H. cydno*) (Figure 2, Table S2, and Table S3). In all cases the former taxon shows the presence of the yellow hindwing bar, which is absent in the latter of each pair. For the *melpomene* vs *rosina* comparison, 21 coding and non-coding regions were sampled, representing a total of 11,578 bp of sequence, for *amaryllis* vs *aglaope* eight regions representing 4085 bp and for *H. pachinus* vs. *H. cydno* 12 regions representing 5596 bp. Between both races and species there was greater genomic divergence across the *HmYb* region as compared to unlinked genes (Table 1). In total 806 variable sites were tested for *HmYb*, generating two genomic regions with significant genotype-by-phenotype association. First, both pairs of *H. melpomene* races showed significant associations around the *HM00007*, *HM00008* and *HM00010* genes (Figure 3, Table S4). Second, the strongest peaks of association between the species *H. cydno* and *H. pachinus* and between *H. m. amaryllis* and *H. m. aglaope* were around the *HM00023* and *HM00024* genes (Figure 3). This was most marked in the comparison between *H. cydno* and *H. pachinus*, which showed 14 significantly associated SNPs in this region (in *HM00024*, the region adjacent to it and a non-coding region near *HM00025*). Such a pattern was not seen at any of 16 unlinked loci studied previously for this species pair [34]. In all cases however, there was a pattern of sites associated with phenotype being interspersed with others showing no or little association.

**Table 1:** Comparison of population differentiation at the *HmYb* locus compared to unlinked loci between species and between races of *Heliconius*.

Comparison	Wing pattern linked loci Mean F*_ST_*	Unlinked loci Mean F*_ST_*	Mann-Whitney test p
*H. m. rosina* vs *H. m. melpomene*-Venezuela	0.197±0.185 (n = 17)	0.043±0.077 (n = 5)	0.013
*H. m. rosina* vs *H. m. melpomene*-Panama	0.0121±0.062 (n = 8)	0.006±0.034 (n = 5)	0.008
*H. m. aglaope* vs *H. m. amaryllis*	0.158±0.078 (n = 5)	0.007±0.002 (n = 3)	0.022
*H. m. aglaope* vs *H. m. amaryllis (HmB)*	0.281±0.196 (n = 8)	0.007±0.002 (n = 3)	0.019
*H. cydno* vs *H. pachinus*	0.380±0.169 (n = 11)	0.136±0.210 (n = 16)	0.004

Values are the mean F*_ST_* values across all the sequenced loci ± standard deviations; n indicates the number of loci included in the analysis.

The two races of *H. melpomene*, that differ in presence of the *HmB* red forewing band, were also studied for *HmB* linked markers [20]. *H. melpomene amaryllis* has a red forewing band, controlled by the dominant *HmB* allele, which is absent in *H. melpomene aglaope* (Figure 2). *H. m. aglaope* and *H. m. amaryllis* were screened for a total of seven genes across the *HmB* region plus the closely linked gene *MRSP*, covering a total of 5391 bp (Table S3). Again, linked *HmB* markers showed a higher overall level of genetic differentiation between races as compared to unlinked genes (Table 1). In total 40 variable SNPs were tested for genotype-by-phenotype association, of which 16 showed a significant association (Figure 3, Table S5). The peak of association was located around the *slu7*, *kinesin* and *GPCR* genes (Figure 3). Several SNPs in these three genes were fixed in *H. m. amaryllis*, with the alternate variant at high frequency (>90%) in *H. m. aglaope*. The lack of completely fixed differences between any of the comparisons suggests that we have not yet sampled causative sites, or that there is epistasis between the variable sites with phenotype determined by allelic combinations.

### Patterns of variation and deviations from neutrality

Selective sweeps act to reduce genetic variability and alter the frequency spectrum of haplotypes. However, interestingly, in contrast to more recently evolved phenotypes such as pesticide resistance, there was no clear signal of recent selection in our data. Levels of nucleotide diversity varied between genes, but with no clear pattern of reduced variation in any population, or around the genomic regions showing genotype-by-phenotype associations (Table S6). There were three adjacent genes in the *H. m. aglaope* population that showed a significantly negative Tajima's D (Table S6), although this region was not in the peak of genetic association with phenotype. Otherwise, there was no consistent pattern across either region, with a few individual genes showing significant deviations from neutrality for particular populations, among markers linked and unlinked to colour pattern loci (Table S6). Overall levels of nucleotide diversity were lower than in the corresponding populations of *H. erato*, consistent with the observed smaller population sizes of *H. melpomene* in the field [27].

### Patterns of linkage disequilibrium

**Figure 4:**
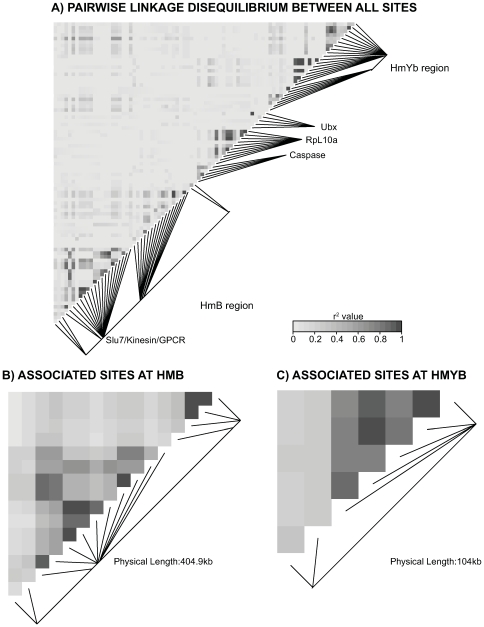
Linkage disequilibrium (LD) for combined *H. m. aglaope* and *H. m. amaryllis* populations. Pairwise LD between sites across the genomic region is shown as a heatmap, with greyscale indicating estimated r^2^ values. Darker colours indicate a stronger correlation between SNP variants at the two sites, across the individuals sampled. The highest LD estimates are all within gene regions, or between closely linked loci. The largest block of LD is across the region with strong genotype-by-phenotype association in the *HmB* region, across the *Slu7*, *Kinesin* and *GPCR* genes (A, lower left). Comparisons are shown for a) all sites, b) sites associated with phenotype in the *HmB* locus, and c) sites associated with phenotype in the *HmYb* locus.

There was, however, good evidence for strong linkage disequilibrium across colour pattern regions, most notably around the three associated genes in the *HmB* region (Figure 4). LD analysis was restricted to the *H. m. aglaope* and *H. m. amaryllis* population samples, which had larger sample sizes (n>20 per population). When LD was plotted against genetic distance, there was strong LD (0.5>r^2^>0.9) between sites separated by a distance of up to 14 kb (Figure S2A). This long distance LD was primarily due to sites associated with phenotype at both *HmB* and *HmYb* – when such sites were removed from the analysis long distance LD virtually disappears (Figure S2B). The only exceptions were a few pairwise comparisons between sites in the *Slu7* and *GPCR* genes with high LD. These are not between sites associated with phenotype, but could presumably have resulted from a history of selection across this region (Figure S2B). The analysis excluding sites associated with phenotype indicates that r^2^ values rapidly decay to a background level below about 0.3 at a distance of around 500 bp. This is further indicated in analysis of LD within genes, where strong linkage disequilibrium was seen up to a distance of around 500 bp, which similarly decayed rapidly (Figure S3). Thus, the background pattern of rapid decay in LD would appear to be similar in *H. melpomene* and *H. erato*, but in *H. melpomene* this is overlaid by long-range haplotype structure that is associated with wing pattern divergence [12].

### Expression analysis of candidate genes

For the *HmB* locus, where our population genetic analysis indicates a clear candidate region, we followed up with analysis of gene expression in developing wing discs. Four genes were chosen for quantitative expression analysis in developing wings, either because they were located in the peak of genetic differentiation (*Slu7*, *Kinesin* and *GPCR*), or as being a nearby candidate locus for pattern specification (*Mad*). The same populations studied above were not available as live material, so we instead used two forms available from commercial suppliers, *H. melpomene malleti* and *H. melpomene cythera*, which have yellow and red forewing bands respectively, providing a similar comparison as that between *H. m. aglaope* and *H. m. amaryllis*. First, forewings were removed from three pupal developmental stages of *H. melpomene cythera*, and dissected into three wing segments, to examine spatial gene expression between proximal, medial (*HmB*) and distal regions (expression analysis “between wing segments”). Second, whole forewings and hindwings were separately dissected from 5^th^ instar larvae and early pupae of *H. melpomene malleti* and *H. melpomene cythera* for expression analysis ‘between races’.

**Figure 5:**
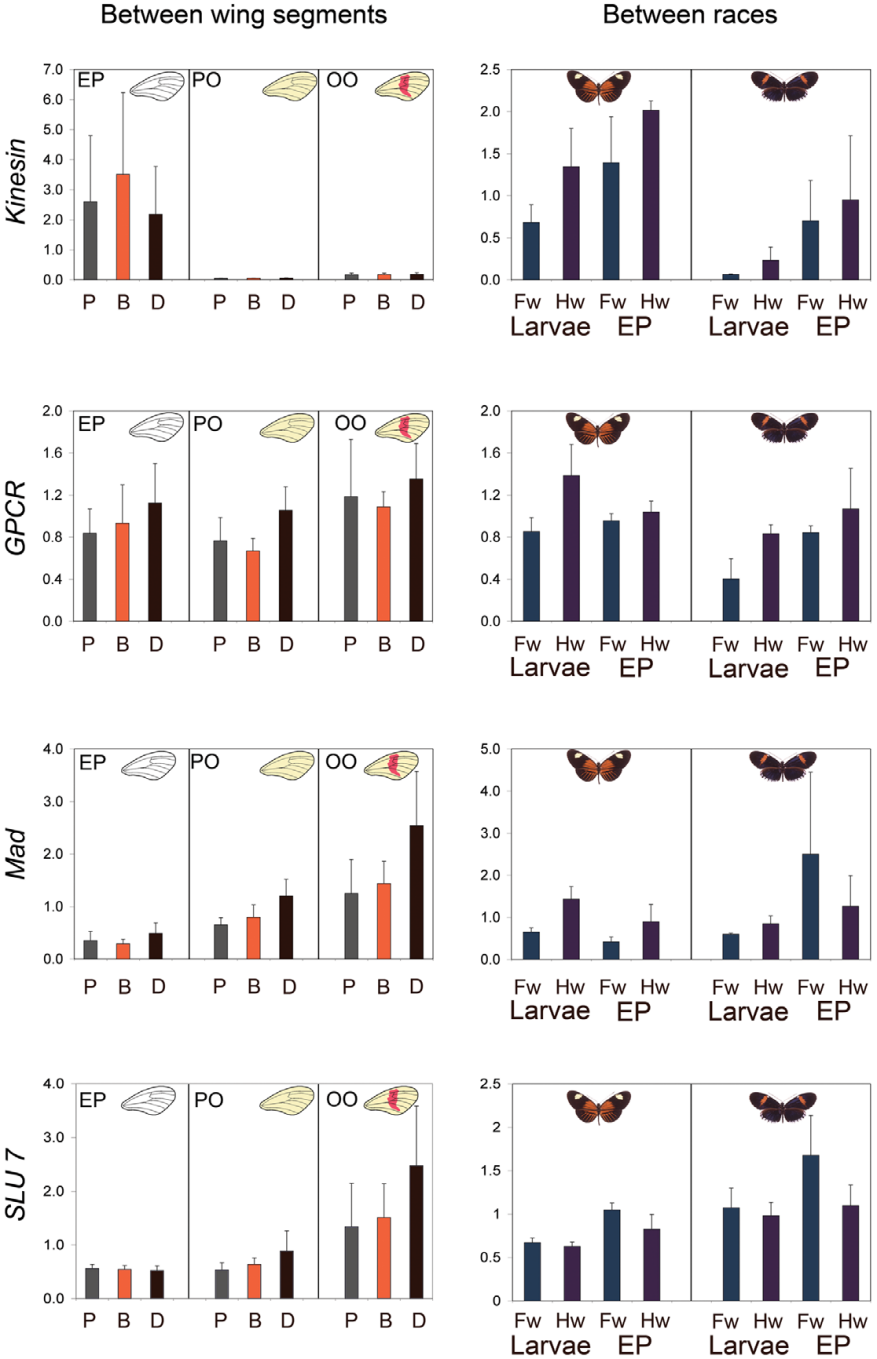
Mean and standard error of relative gene expression for *Mad*, *Slu7*, *Kinesin*, and *GPCR*. For the ‘Between wing segments’ experiment, *H. melpomene cythera* forewings were dissected into P (proximal), B (band) and D (distal) segments of three pupal developmental stages; EP (early pupae), PO (pre-omochrome) and OO (only omochrome). For the “Between Races” experiment, forewings (Fw) and hindwings (Hw) of *H. m. malleti* (left side) and *H. m. cythera* (right side), were dissected from Larvae (5^th^ instar) and EP (early pupae).

The most striking result was a strong contribution to the prediction of gene expression by race at the *kinesin* gene, with *H. m. malleti* having an overall higher expression than *H. m. cythera* (Figure 5). Analysis using Bayesian Model Averaging gave Pr(β≠0) = 66.5, indicating a probability of 66.5% that the coefficient of variation for race in this experiment was greater than zero. The fold-change in expression across all samples represented 2.6 times greater expression in *H. m. malleti* as compared to *H. m. cythera*. In the ‘between wing segments’ experiment, the *kinesin* gene showed higher expression in the early pupal (EP) stage but no differences between different segments of the wing, consequently developmental stage made a strong contribution to gene expression prediction (Pr(β≠0) = 89.6; Figure 5).

In the ‘between wing segments’ experiment, *Slu 7* showed higher expression in later developmental stages (highest in the ommochrome only stage [OO]), but no differences between segments of the wing (developmental stage Pr(β≠0) = 100). Race made a contribution to the prediction of gene expression in the ‘between races’ experiment (Pr(β≠0) = 86.5), with *H. m. cythera* having a 1.52-fold overall higher expression than *H. m. malleti* (Figure 5). No main variables made a contribution to *GPCR* gene expression in either ‘between wing segments’ or ‘between races’ experiments. The *Mad* gene showed an increase in expression levels during pupal development, with highest expression at the latest stage surveyed (developmental stage Pr(β≠0) = 94), but no differences between races or wing segments (Figure 5).

## Discussion

The loci controlling adaptive radiation in *Heliconius* are genomic ‘hotspots’ for evolutionary change and speciation [8],[10],[35]. Here, and in a companion paper in this issue, we have carried out the first surveys of population variation across two such chromosomal regions in the co-mimic species, *H. melpomene* and *H. erato* [12]. In *H. melpomene*, there are strong peaks of genotype-by-phenotype association in both the *HmYb* and *HmB* regions, and strong linkage disequilibrium between associated sites at distances of up to 14 kb. Significant genotype-by-phenotype association and long distance LD provide striking evidence for the influence of strong selection on the genome. However, despite strong selection, there was no signal of a classic ‘selective sweep’, consistent with a long history of recombination during the spread of these alleles. Furthermore, we identify a strong candidate for the *HmB* locus, a kinesin gene that shows parallel patterns of upregulation in the yellow forewing banded races of both *H. melpomene* and *H. erato*.

### Genetic differentiation between races

Hybrid zones in *H. melpomene* separate forms that are only differentiated at a few major colour pattern loci. Such zones provide a powerful system for identifying adaptive genetic changes. Genes under selection are strongly differentiated, but there is only a weak barrier to gene flow at the rest of the genome [22]. This is confirmed by the lack of genetic differentiation at loci unlinked to colour pattern seen in our data. In contrast, there was increased genetic differentiation across the candidate regions in all cases (Table 1), implying genetic hitch-hiking around sites under colour pattern selection. At the *HmYb* locus, levels of differentiation are greater between reproductively isolated species, as compared to races, consistent with higher overall levels of reproductive isolation and ‘divergence hitch-hiking’ between species [36],[37].

Across both genomic regions, there were clear peaks of genotype-by-phenotype association. Our sampling of multiple phenotypic comparisons at the *HmYb* locus offers the first comparative analysis of parallel phenotypic evolution in *Heliconius*, and indicates that at a fine scale the genes responsible for parallel changes in phenotype are shared, but not identical across different hybrid zones. Most strikingly, the region around *HM00024* (*LRR-3*) shows a very marked peak in both the *H. pachinus* vs. *H. cydno* and the *H. m. amaryllis* vs *H. m. aglaope* hybrid zones, and is also highlighted in *H. erato* [12]. The genes in this region are therefore the most strongly implicated in regulation of wing pattern for *HmYb*. In addition however, another peak around *HM00008* and *HM00010* is shared between the two *H. melpomene* inter-racial comparisons, but not with *H. erato*. The repeatability of these patterns across independent hybrid zones suggests that multiple associations are not merely the stochastic results of selection at a single causative site, but rather imply a role for multiple functional sites underlying wing pattern differences. This is supported by the observation of increased linkage disequilibrium between associated sites at the *HmYb* locus as compared to background levels, suggesting that selection is maintaining particular allelic combinations across this region (Figure S1).

The comparison across the *HmB* region also gave a clear peak of genotype-by-phenotype association near three genes, *kinesin*, *GPCR* and *Slu7*. In this case the association was stronger than between races at the *HmYb* locus, with several SNPs across these three genes being completely fixed in *H. m. amaryllis* and with a frequency difference of >95% between races. This is also a much stronger signal of differentiation than seen in comparisons between races of *H. erato* at this locus [12]. Furthermore, unlike the pattern in *H. erato*, there was evidence for extensive LD between associated sites up to 14 kb apart. As at *HmYb*, against a background of estimated r^2^ values below 0.3, the long-range LD at *HmB* similarly suggests a haplotype structure maintained by selection.

### Lack of a recent selective sweep

However, in neither genomic region studied here was there consistent evidence for a recent selective sweep in any of the populations. This is perhaps to be expected given the age of the *H. melpomene* radiation (≈250,000–500,000 years [38]), and contrasts with the pattern seen in very recently evolved traits such as pesticide resistance. Similar to *H. erato*, levels of genetic variation showed no consistent pattern between linked and unlinked loci. Although three adjacent genes did show a significantly negative Tajima's D at the *HmB* locus, these did not correspond to the region of strong population differentiation, suggesting no strong deviations from neutrality due to wing pattern selection. Furthermore, both LD and genotype-by-phenotype analyses indicated that sites associated with phenotype were interspersed with variable sites showing no such association. The data are therefore consistent with the wing patterning alleles of both *H. melpomene* and *H. erato* being relatively ancient, such that considerable genetic variation has arisen subsequent to any initial selective sweep. An alternative is that the wing pattern alleles spread in a ‘soft’ sweep, with an initial period in which the novel allele was found at low frequency for an extended period, giving ample opportunity for recombination around selected sites [39]. Thus, the peaks of genetic differentiation seen between races are consistent with the strong selection known to act on wing pattern [29], but have presumably been associated with a long history of hybridization and recombination between the races such that any signal of a recent selective sweep has been erased.

The results therefore appear to support the ‘shifting balance’ model for the evolution of *Heliconius* colour pattern races [40], whereby novel wing patterns arise and spread through otherwise continuous populations behind moving hybrid zones [41]. The ‘Pleistocene refuge’ model seems less likely, as recent contact after extended periods of geographic isolation would presumably have left a stronger signal of genetic differentiation between divergent races, perhaps across the genome but especially more strongly in regions linked to patterning loci [42].

Overall, the stronger signal of both haplotype structure and genotype-by-phenotype association in *H. melpomene*, as compared to *H. erato*, is also consistent with what is known of their biology. First, *H. erato* tends to be more widespread and have larger population sizes [27], leading to higher levels of recombination and a more rapid breakdown of linkage disequilibrium generated by selection. Second, *H. melpomene* is thought to be the mimic of *H. erato*, such that the pattern alleles may have arisen more recently in the former. Nonetheless, in both species the genetic divergence is highly localised when compared to other recent examples, such as ecological races of the pea aphid *Acyrthosiphon pisum*, where islands of divergence extend several cM around QTL under divergent selection [36], or freshwater races of sticklebacks where linkage disequilibrium is extensive around the locus controlling dermal plate morphology [5]. The data are perhaps more similar to that seen in two ecomorphs of the inter-tidal snail, the Rough periwinkle (*Littorina saxatilis*), where regions of differentiation are limited to a few hundred base pairs [37]. High recombination and localised islands of divergence mean that in the future, population genetic analysis is likely to be a powerful tool for isolating functional sites in *Heliconius*.

### Identifying the functionally relevant genes

The development of scale-covered wings, pigmentation and an elaborate patterning system are evolutionary innovations of the Lepidoptera and have been cited as an example of the redeployment of conserved gene networks [43]. Notably, both the *hedgehog* and *wingless* signalling pathways are involved in wing eyespot formation [44],[45]. Similarly, studies of *Drosophila* wing pigmentation have implicated cis-regulatory regions of shared pigment biosynthesis genes in regulating inter-specific differences [46],[47]. However, no obvious members of either canonical signalling or pigment biosynthesis pathways are present in the *HmB* region, similar to the pattern already found in *HmYb* [33]. However, in contrast to a candidate gene approach, the linkage mapping method we have taken makes no *a priori* assumptions regarding the identity of the wing patterning genes. Instead we have cloned the genetic region controlling natural variation in a striking phenotype, and in doing so ruled out a role for any of the wing pattern ‘toolkit’ genes described previously. Of course, these two approaches may be complementary, and the locus that is controlling the wing phenotype may be an upstream regulator of classic ‘toolkit’ genes. Indeed, the intriguing observation that the ‘*Bigeye*’ pattern mutant of *Bicyclus anynana* maps to the homologous chromosome of *HmYb* might indicate that the gene networks uncovered in *Heliconius* may have a general role in butterfly wing patterning [48].

At the *HmYb* locus, two regions are implicated, first around the genes *HM00007*, *HM00008* and *HM00010*. These genes have putative orthologues in *Drosophila* (*CG14870/B9 protein*, *CG5098* and *CG3184*) about which little is known, except that *HM00010* contains WD40 repeats that are likely to be involved in protein-protein interaction. More striking is the association found around *HM00024* (*LRR-3*), which was replicated in two of the comparisons studied here and in *H. erato*. This gene shows similarity to *Sur-8* in *Drosophila*, consistent with a possible role in signal transduction, and is also adjacent to *HM00023*, a probable non-coding transcript with complex patterns of alternative splicing that may have a regulatory function [33]. Overall, therefore, the coding regions implicated do not show any homology to well characterized genes in other species. The only candidate gene for *HmYb* suggested by sequence similarity was the transcription factor *Unkempt* which, unlike in *H. erato*, showed no significant association with wing pattern in *H. melpomene*.

Against a background of multiple associated sites, and extensive LD across three genes, there is one strong candidate for the *HmB* gene. In both *H. melpomene* and *H. erato* when yellow and red forewing band forms are compared, the former showed much higher expression levels of the putative kinesin, *HM01018*, in late larval and early pupal stages, suggesting a role for this locus in wing pattern specification. Members of the kinesin superfamily play diverse roles in cellular organization, using a catalytic motor to move along microtubules and transport organelles and molecules [49]. Homology predicts the *H. melpomene kinesin* contains an N-terminal motor domain, yet has no sequence similarity to other eukaryotes in the C-terminal cargo domain, making it difficult to speculate regarding the function of this gene. In *Drosophila*, kinesin proteins can function to determine cell polarity and RNA localisation during embryogenesis, and are therefore known to play a role in pattern specification at a cellular level [50]. Furthermore, movement of melanocytes in vertebrate pigmentation involves kinesin molecules [51], and there has been speculation regarding a possible role in invertebrates [52]. Localisation of pigments, or pigment precursors in cellular vesicles might similarly be involved in butterfly wing pigmentation. It therefore seems plausible that increased levels of the *kinesin* expression across the whole wing regulate either scale cell fate or pigment localization, and thus ultimately specify pattern formation.

### Evolution of a genomic hotspot

Our data shows that adaptive changes in *Heliconius* are clustered in the genome across multiple independent hybrid zones. This adds to what is already known from crossing experiments, showing that two patterning loci, *HmB* and *HmD*, controlling the red forewing band and orange hindwing rays respectively, are tightly linked, as are *HmYb* and *HmSb* that similarly control the yellow hindwing bar and white margin [31]. There is also evidence for genetic associations between these colour pattern loci and other adaptive traits, including mate and host plant preference [53][R. Merrill and C. Jiggins, Unpub.]. Thus, these regions are functional ‘hotspots’ in the *Heliconius* genome that play a disproportionately large role in pattern specification and indeed, in speciation. The population data described here offer novel insights into the genetic architecture of these regions. First, there is significant association of both genetic variation and long distance haplotype structure with wing pattern at both loci, indicating the influence of strong mimicry selection on the genome. Nonetheless, consistent with previous estimates of the age of *H. melpomene* (≈250,000–500,000 years [38]), there is no evidence for a recent selective sweep involving reduced variation or significant deviations from neutrality. This might be an indication that these alleles are indeed part of an ancestral ‘toolkit’ of wing patterning variants that are shared between taxa through hybridization [54], or simply that there has been sufficient time since novel variants arose for the signal of a selective sweep to have been erased. This pattern may be more representative of most natural adaptation, which has arisen over millions of years, as compared to recently derived traits under strong man-made selection, such as pesticide resistance or domestication. In summary, our analysis of the population genetics of these regions offers novel insights into the evolutionary history of a spectacular parallel adaptive radiation.

## Methods

### BAC sequencing and annotation

A BAC tile path containing seven clones was constructed across the *HmB* locus [31] and sequenced by the Wellcome Trust Sanger Institute to HTG phase 3 quality; AEHM-22C5 (160673 bp) CU462842, AEHM-7G5 (179057 bp) CU462858, AEHM-28L23 (125911 bp) CU467808, AEHM-27I5 (126643 bp) CU467807, AEHM-19L14 (136956 bp) CU672261, AEHM-21P16 (129716 bp) CU681835 and AEHM-28F19 (122304 bp) CU672275. Linkage mapping has shown that the *HmD* locus overlaps the *HmB* locus, and extends beyond clone AEHM-22C5. A gap in the BAC library prevented this region being fully sequenced so we cannot be sure that *HmD* is among the loci studied here. For sequencing and annotation of the *HmYb* locus, see Ferguson et al. [33].

BAC clones in the *HmB* tile path were individually annotated with 454-EST contigs and a repeat database, using the annotation pipeline MAKER [32], which identifies repeats, aligns ESTs and proteins to a DNA sequence, produces *ab initio* gene predictions, and automatically synthesizes the data into gene annotations having evidence-based quality indices. The 454-EST contigs were generated from normalised cDNA from wing disc mRNA of two *H. melpomene* races and sequenced using 454 FLX (48Mb for *H. m. malleti* and 103Mb for *H. m. cythera* [33]). Genome sequence repeat motifs were characterized by screening 32,528 *H. melpomene* BAC end sequences (generated by Sanger di-deoxy sequencing) with the ReRep pipeline [55], which is specifically optimised for identifying repetitive structures in a genome survey sequence (GSS) dataset. BLAST searches were performed against the non-redundant UniRef100 database, and gene predictions were generated using SNAP (Semi-HMM-based Nucleic Acid Parser) [56]. The resulting gff files from the MAKER pipeline were analyzed and viewed with the Apollo Genome Annotation Curation Tool [57] version 1.9.6. (Table S2).

### Population samples

Adult butterflies were collected, wings removed and bodies preserved in 20% DMSO, 0.2 M EDTA salt saturated solution. Genomic DNA was extracted using DNeasy blood and tissue kit (Qiagen). *H. cydno galanthus* (n = 33) and *H. pachinus* (n = 22) were collected from Costa Rica; *H. melpoomene melpomene* (n = 16) and *H. melpomene rosina* (n = 19) were collected from Panama and Venezuela; *H. melpomene aglaope* (n = 30) and *H. melpomene amaryllis* (n = 30) were collected from Peru (Figure 1). Two admixed individuals were included in the sample for *H. melpomene amaryllis* (see Figure S4). The Peruvian race *H. m. aglaope* is phenotypically almost identical to the Ecuadorean form *H. m. malleti* used in transcriptome sequencing.

### Gene sequencing

PCRs contained 10–50 ng of genomic DNA, 1× reaction buffer, 2.0 mM MgCl_2_, 0.1 mM dNTP, 50 pmol of each primer, 0.25 units of Taq polymerase (Bio-Line). Thermal cycling conditions were 94°C 1 min, 35 cycles of 94°C 15 sec, annealing 30 sec, 72°C 60 sec, and a final extension of 72°C. Prior to sequencing, products were incubated with 2 units of Exonuclease 1 (NEB) and 1 unit of Shrimp Alkaline Phosphatase (Fermentas) for 40 minutes at 37°C then 80°C for 20 minutes. Sequencing was performed in 10 µl reactions using 1–3 µl of template, 1× reaction buffer, 1µl BigDye terminator v3.1 (Applied Biosystems) and 0.32 pmol primer and run in a thermal cycler for 25 cycles of 95°C 30 seconds, 50°C 20 seconds, 60°C 4 minutes. Products were then sequenced using an ABI3730 capillary sequencer, analyzed using CodonCode Aligner software and single nucleotide polymorphisms identified manually. Genes contained insertion/deletion (IN/DEL) variation were trimmed to exclude such regions from analysis, or cloned using Promega pGEM-T Easy kit before sequencing (Table S1).

Fragments of seven genes across the *HmB* region, plus the linked gene *MRSP* (approx. 1 cM from the *HmB* locus), were sequenced, along with three control genes unlinked to wing colour patterning loci (Table S1). In total, 19 genomic fragments across the *HmYb* candidate region and three fragments linked to *HmYb*, but outside the candidate region, were amplified from at least 5 individuals from one or more of the pairs of hybridising subspecies/species. In addition 3–5 unlinked genes were sequenced for each pair of hybridizing *H. melpomene* subspecies.

### Population genetics

Haplotypes were inferred using Phase/Unphase implemented in DnaSP [58]. Further population genetic analysis was carried out in DnaSP including calculation of nucleotide diversity (π) [59], Tajima's D for each population using all segregating sites [60], and F*_ST_* between colour pattern races at each locus [61]. The statistical significance of Tajima's D estimates was estimated using the two-tailed test assuming D follows a beta-distribution as proposed by Tajima and implemented in DnaSP [60]. Samples of the race *H. m. melpomene* collected from distant localities in Panama and Venezuela were analyzed separately and compared independently with *H. m. rosina*. Comparisons between F*_ST_* values for genetic markers within the colour pattern regions (as defined by the linkage study) and unlinked markers were carried out for each pair of populations using a Mann-Whitney *U* test implemented in Minitab v15.1 (Minitab Inc.). For the *H. cydno/H. pachinus* comparison background levels of differentiation were obtained from 16 previously published genes [34].

We determined if any SNPs were associated with a colour pattern phenotype using a chi-squared linear trend test [62],[63]. This test assumes a linear relationship between the phenotype and genotype and applies a chi-square goodness-of-fit test to determine if the genotype at a SNP is significantly associated with a particular wing colour pattern. In total 679 variable sites were tested for *HmYb* in *H. melpomene*, 127 in *H. cydno/pachinus*, 40 variable sites for *HmB* between *H. m. aglaope* and *H. m. amaryllis* and 20 sites at unlinked loci. Colour pattern genotypes at the *HmYb* and *HmB* loci were scored as 0.0 or 1.0 representing alternative homozygotes, and 0.5 for heterozygotes. Although *HmB* is dominant, two hybrid individuals with the dominant red forewing *HmB* allele and orange rayed *HmD* alleles were scored as heterozygotes, as recombinants between *HmB* and *HmD* are rare. A stringent significance cut-off was calculated using a Bonferroni correction applied to all 866 sites tested (−Log_10_(0.05/866) = 4.24).

Linkage disequilibrium across *HmYb* and *HmB* was calculated for all population samples with a sample size ≥20. This restricted the analysis to the *H. m. aglaope* and *H. m. amaryllis* populations, and gave a total of 40 sites for *HmB*, 24 for *HmYb* and 20 for unlinked loci with a rare allele frequency greater than 0.05 that were considered informative for LD analysis. Multi-allelic sites that had a minor allele with a frequency less than 0.05 were condensed to bi-allelic SNPs by merging the minor allele genotypes. LD was estimated using the commonly used composite estimate of LD method described by Weir [62], which does not assume HWE or that haplotypes are known (see also [12]). The R package LDHeatmap was used to visualize LD across both regions [64]. LD estimates are presented for combined *H. m. aglaope* and *H. m. amaryllis* populations.

### Quantitative gene expression

Quantitative real time PCR (qPCR) was used to determine the relative transcript levels of *Mad*, *slu7*, *kinesin* and *GPCR*. RNA was extracted from forewings of *H. m. cythera* pupae at three developmental stages; EP (Early Pupae ∼48 hours after pupation, poorly developed wings, no veins), PO (Pre-Ommochrome – well developed wings with veins but no pigmentation) and OO (Only-Ommochrome – red band visible but no melanin present) [65]. Each of these forewings was dissected into three sections to compare gene expression between wing regions (P = proximal, B = region that develops the red *HmB* band and D = distal) totalling 27 samples (3 individuals per stage ×3 developmental stages ×3 wing segments).

To compare pattern races we used *H. m. cythera* and *H. m. malleti* individuals. The former has a red forewing band, which is absent in the latter. RNA was collected from whole forewings and hindwings of three individuals from two developmental stages (late 5^th^ instar larvae and EP), totalling 24 samples (3 individuals ×2 wings ×2 developmental stages ×2 races).

Total RNA (500 ng) was reverse transcribed with random hexamers and 1 µl of the resulting cDNA template (25ng) was combined with 200 nM of each primer in 25 µl of total reaction volume containing 1× SYBR Green master mix (*SensiMix*, Quantace). The reactions were subjected to 40 cycles of amplification in an Opticon 2 DNA engine (MJ Research) under the following conditions: 95°C for 15 min and then 40 cycles of 94°C for 15 sec, 55.4°–60°C for 30 sec, 72°C for 30 sec followed by a final incubation at 72°C for 10 min. Melting curves were generated between 55° and 90° with readings taken every 0.2° for each of the products to check that only a single product was generated. We sequenced at least one product from each set of primers to confirm identity and size. For most loci, the amplified fragments spanned at least one intron, ensuring that genomic DNA contamination could be identified. We used two housekeeping control genes for normalization in the ‘between wing segments’ experiment; *EF1-α* and *RpS3A*. However, as results of these two genes were consistent, only *EF1-α* was used in the ‘between race’ experiment. qPCRs on target and control loci were always assayed using product from the same cDNA synthesis reaction. Furthermore all samples were assayed 3 times but, because differences in gene expression between replicates were always <0.05× (see below), we report only the average value for each sample. Statistical methods for qPCR experiments are given in Text S1.

## Supporting Information

Figure S1 Alignment of *HmB* region BAC clones between *H. melpomene* and *H. erato*. To assess gene order using finished BAC sequences, *H. melpomene* clone 28L23 was compared to finished *H. erato* clone 31N19 as well as the homologous region from the recently sequenced genome of the silkmoth, *Bombyx mori*. Gene order and orientation across the *H. melpomene HmB* locus was conserved between the two *Heliconius* species, however, when compared to the silkmoth *Bombyx mori*, there was an inversion containing three genes; *Slu7*, sorting nexin and Phosphodiesterase 10A. The Artemis Comparison Tool software (release 7) was used to compare three sequences; *Bombyx mori* nscaf3026 1161033.1269872 (http://silkworm.genomics.org.cn), *H. melpomene* BAC clone 28L23 (CU467808) and *H. erato* BAC clone 31N19 (accession). Comparisons were performed using tBLASTx, limited to 200 HSPs and an expect value 1.0e-10. *B. mori* genes displayed are BGIBMGA011292-TA (*bves*), BGIBMGA011315-TA (Hypothetical protein HM010022), BGIBMGA011316-TA (*Slu7*), BGIBMGA011317-TA (Sorting Nexin), BGIBMGA011291-TA (phosphodiesterase 10A), BGIBMGA011289-TA and BGIBMGA011290-TA (predicted *Kinesin*), BGIBMGA011318-TA (GPCR), BGIBMGA011288-TA (hypothetical protein, not shown), BGIBMGA011287-TA (Epoxide Hydrolase).
(0.15 MB PDF)

Figure S2 Decay of linkage disequilibrium with distance across the two regions. Data are shown for *HmB* (red circles) and *HmYb* (green triangles) for combined *H. m. amaryllis* and *H. m. aglaope* populations, both for all sites (A) and with sites showing a significant association with phenotype removed (B). The only remaining comparisons showing long-range LD after removal of associated sites are also between the *Slu7* and *GPCR* genes, in the region associated with the *HmB* phenotye.
(0.54 MB PDF)

Figure S3 Decay of linkage disequilibrium with distance within gene markers. Data are shown for combined *H. m. amaryllis* and *H. m. aglaope* populations, both for coding and non-coding markers in regions linked and unlinked to colour pattern.
(0.22 MB PDF)

Figure S4 Phenotypes of two hybrid individuals sampled in the Peruvian hybrid zone. Both individuals are phenotypically most similar to *H. m. amaryllis*, but with expression of the dominant *HmD* allele from the Amazonian race controlling the red hindwing rays and basal red patch on the forewing.
(1.62 MB PDF)

Table S1 Annotation of genes in the *HmB* region.
(0.12 MB DOC)

Table S2 Gene sequencing and quantitative PCR primers.
(0.40 MB DOC)

Table S3 Summary of gene regions sequenced for population genetic analysis.
(0.12 MB DOC)

Table S4 *HmYb* SNP data. Details of all sites significantly associated with phenotype at the *HmYb* locus.
(0.05 MB XLS)

Table S5 *HmB* and unlinked gene SNP data. All variable SNPs used in the analysis for the aglaope/amaryllis comparison at the *HmB* locus and at unlinked loci.
(0.05 MB XLS)

Table S6 Estimates of Tajima's D and nucleotide diversity estimates for each locus by population.
(0.28 MB DOC)

Text S1 Supplementary methods.
(0.03 MB DOC)
