# Supplementary figures and images for: Genomic Hotspots for Adaptation: The Population Genetics of Müllerian Mimicry in the Heliconius melpomene Clade
Source: PLoS Genet. 2010 Feb 5;6(2):e1000794. doi: 10.1371/journal.pgen.1000794 (PMC2816687; doi:10.1371/journal.pgen.1000794)

*Bombyx mori* nscaf3026 (1161 kb - 1279 kb)

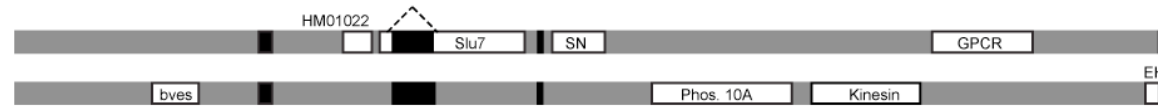

*Heliconius melpomene* BAC clone AEHM-28L23

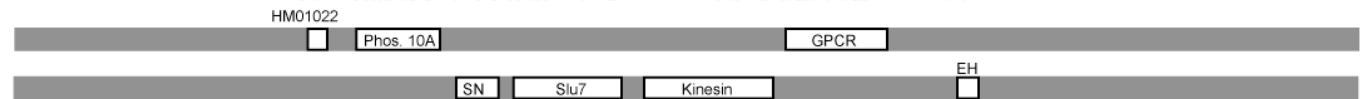

*Heliconius erato* BAC clone 31N19

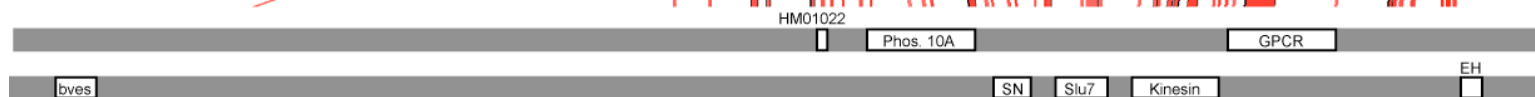

20 kb

Supplement: Figure S1 — Alignment of HmB region BAC clones between H. melpomene and H. erato. To assess gene order using finished BAC sequences, H. melpomene clone 28L23 was compared to finished H. erato clone 31N19 as well as the homologous region from the recently sequenced genome of the silkmoth, Bombyx mori. Gene order and orientation across the H. melpomene HmB locus was conserved between the two Heliconius species, however, when compared to the silkmoth Bombyx mori, there was an inversion containing three genes; Slu7, sorting nexin and Phosphodiesterase 10A. The Artemis Comparison Tool software (release 7) was used to compare three sequences; Bombyx mori nscaf3026 1161033.1269872 (http://silkworm.genomics.org.cn), H. melpomene BAC clone 28L23 (CU467808) and H. erato BAC clone 31N19 (accession). Comparisons were performed using tBLASTx, limited to 200 HSPs and an expect value 1.0e-10. B. mori genes displayed are BGIBMGA011292-TA (bves), BGIBMGA011315-TA (Hypothetical protein HM010022), BGIBMGA011316-TA (Slu7), BGIBMGA011317-TA (Sorting Nexin), BGIBMGA011291-TA (phosphodiesterase 10A), BGIBMGA011289-TA and BGIBMGA011290-TA (predicted Kinesin), BGIBMGA011318-TA (GPCR), BGIBMGA011288-TA (hypothetical protein, not shown), BGIBMGA011287-TA (Epoxide Hydrolase). (0.15 MB PDF) [file pgen.1000794.s001.pdf]

a) All sites

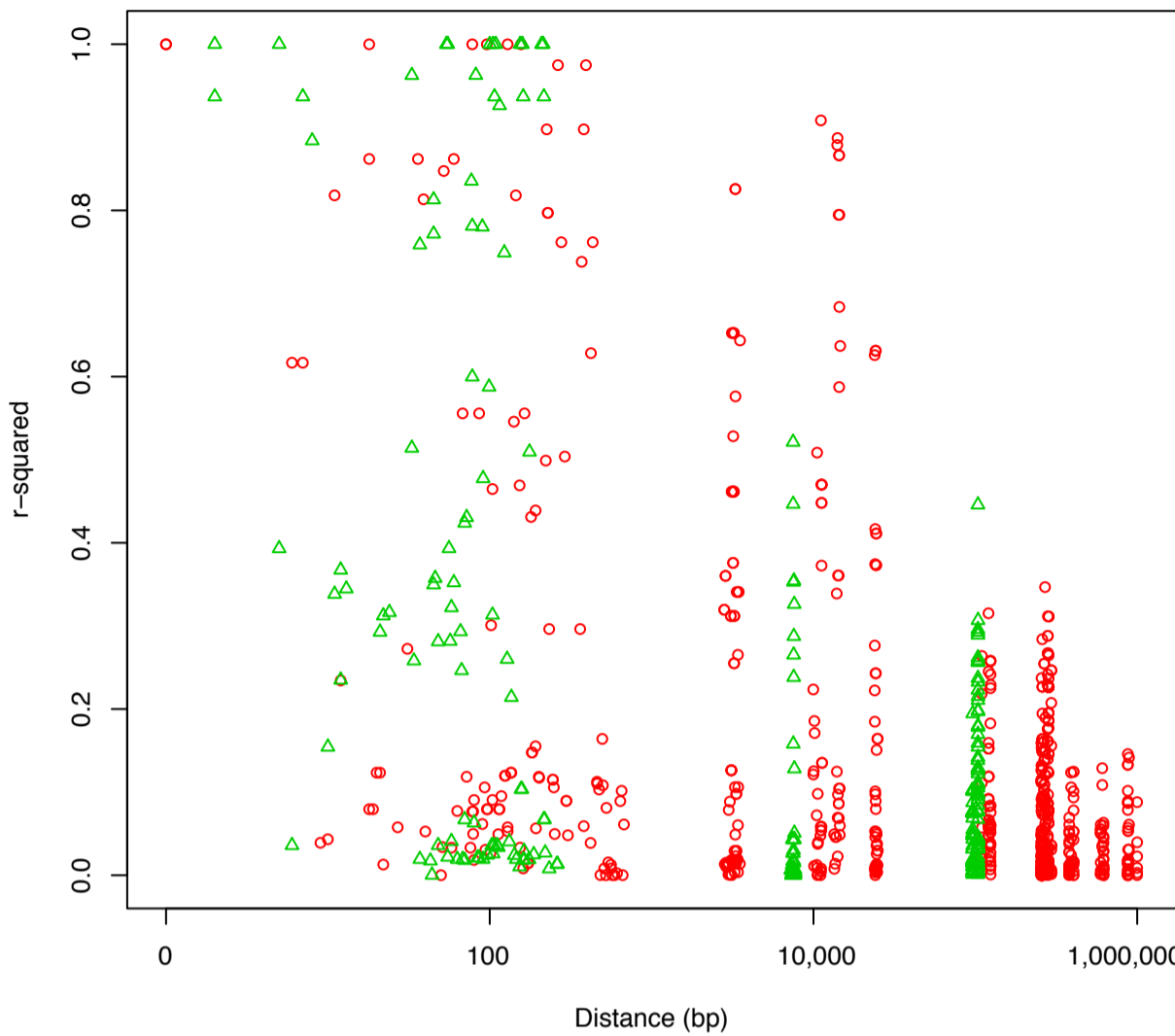

b) Excluding sites associated with phenotype

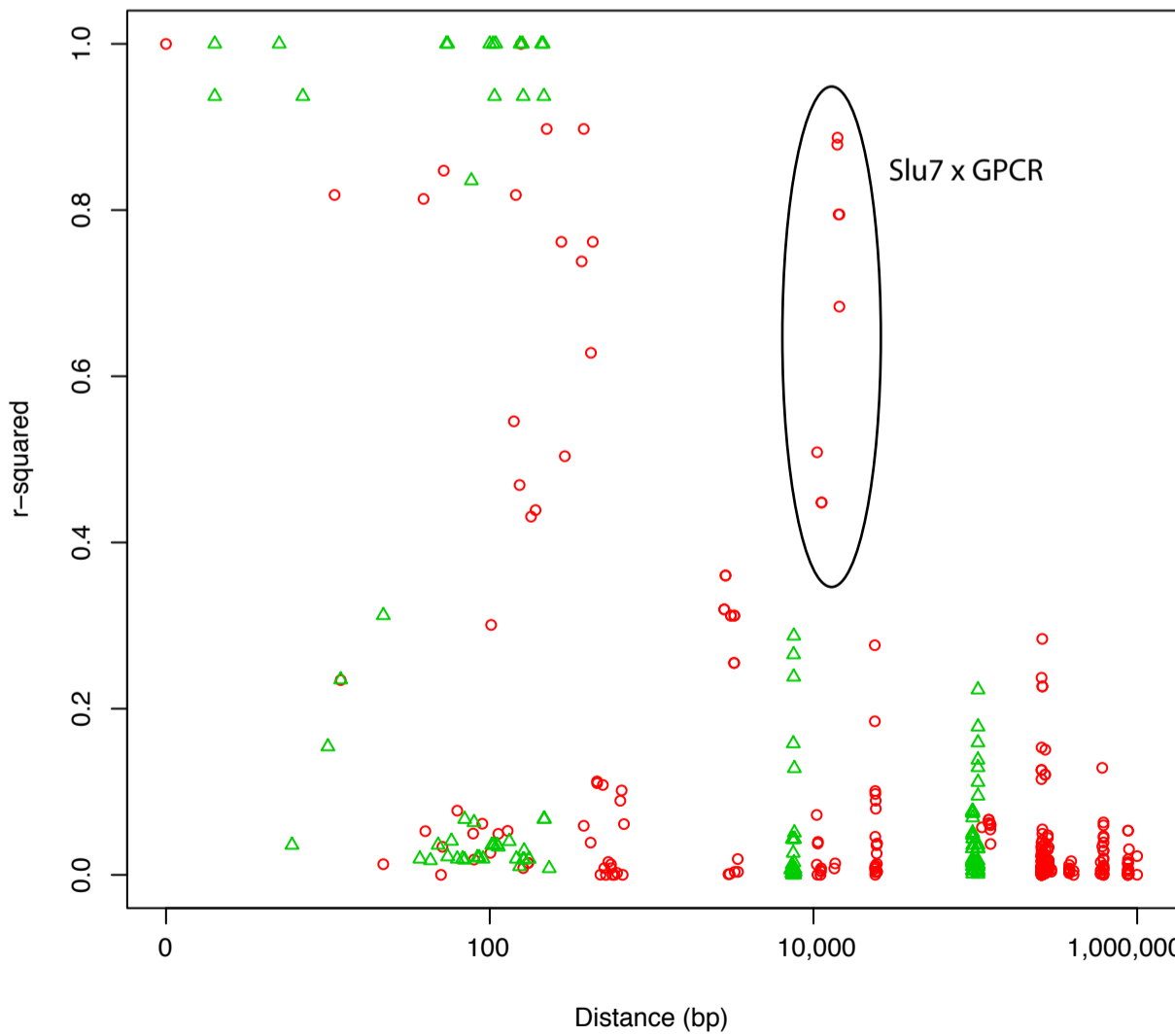

Supplement: Figure S2 — Decay of linkage disequilibrium with distance across the two regions. Data are shown for HmB (red circles) and HmYb (green triangles) for combined H. m. amaryllis and H. m. aglaope populations, both for all sites (A) and with sites showing a significant association with phenotype removed (B). The only remaining comparisons showing long-range LD after removal of associated sites are also between the Slu7 and GPCR genes, in the region associated with the HmB phenotye. (0.54 MB PDF) [file pgen.1000794.s002.pdf]

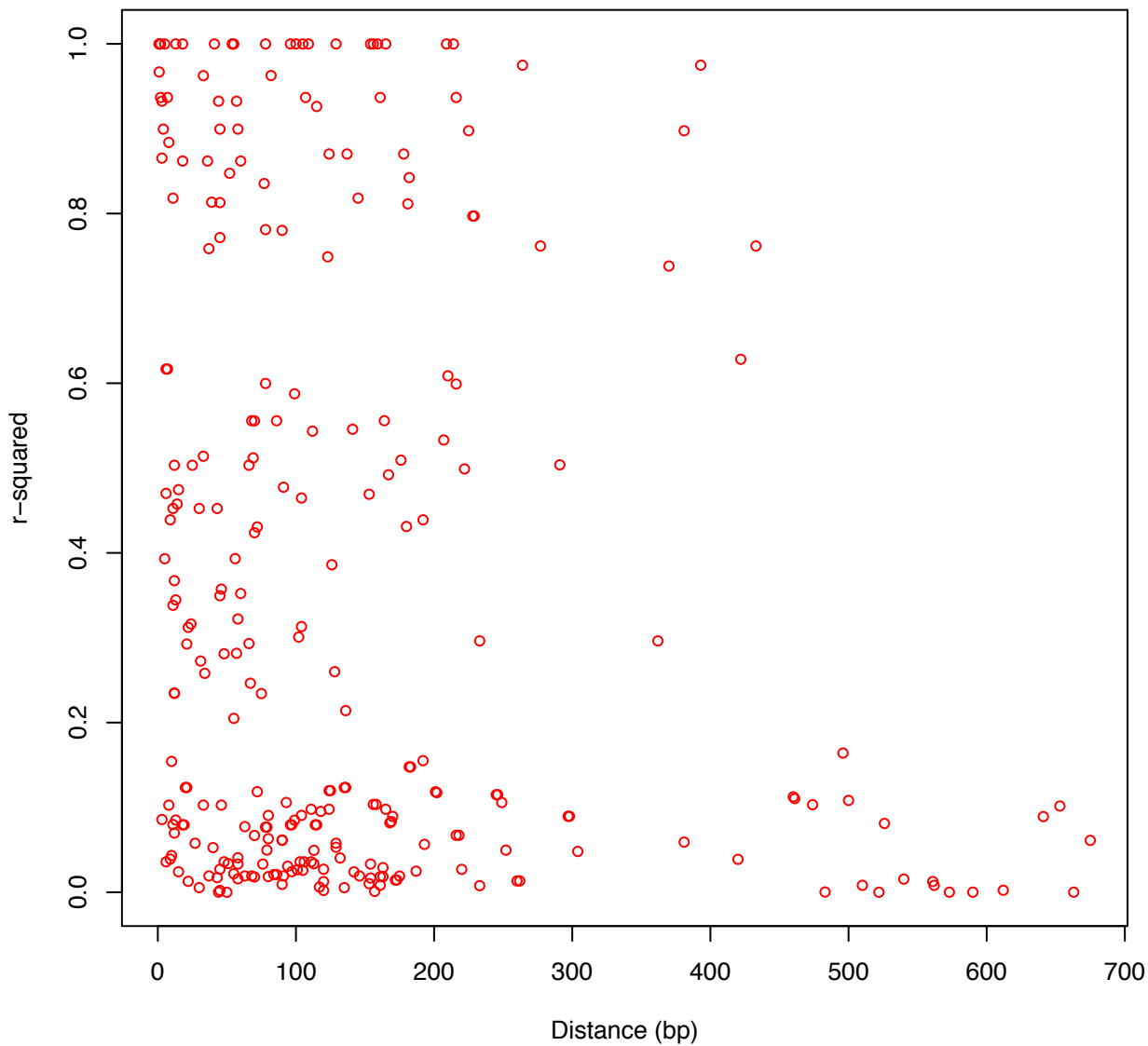

Supplement: Figure S3 — Decay of linkage disequilibrium with distance within gene markers. Data are shown for combined H. m. amaryllis and H. m. aglaope populations, both for coding and non-coding markers in regions linked and unlinked to colour pattern. (0.22 MB PDF) [file pgen.1000794.s003.pdf]
