# Supplementary material for: Genomic Hotspots for Adaptation: The Population Genetics of Müllerian Mimicry in the Heliconius melpomene Clade
Source: PLoS Genet. 2010 Feb 5;6(2):e1000794. doi: 10.1371/journal.pgen.1000794 (PMC2816687; doi:10.1371/journal.pgen.1000794)

Hybrid Individual 1272
6° 27' 07 S, 76° 20' 46 W
Rio Shilcayo, Tarapoto, Peru
Phenotype *Hm B/-,D/-, ybyb, NBNB*

Hybrid Individual 2161
6° 36' 57 S, 76° 11' 10 W
Chumia, Km 14, Shapaja - Chazuta, Peru
Phenotype *Hm B/-,D/-, ybyb, NBNB*

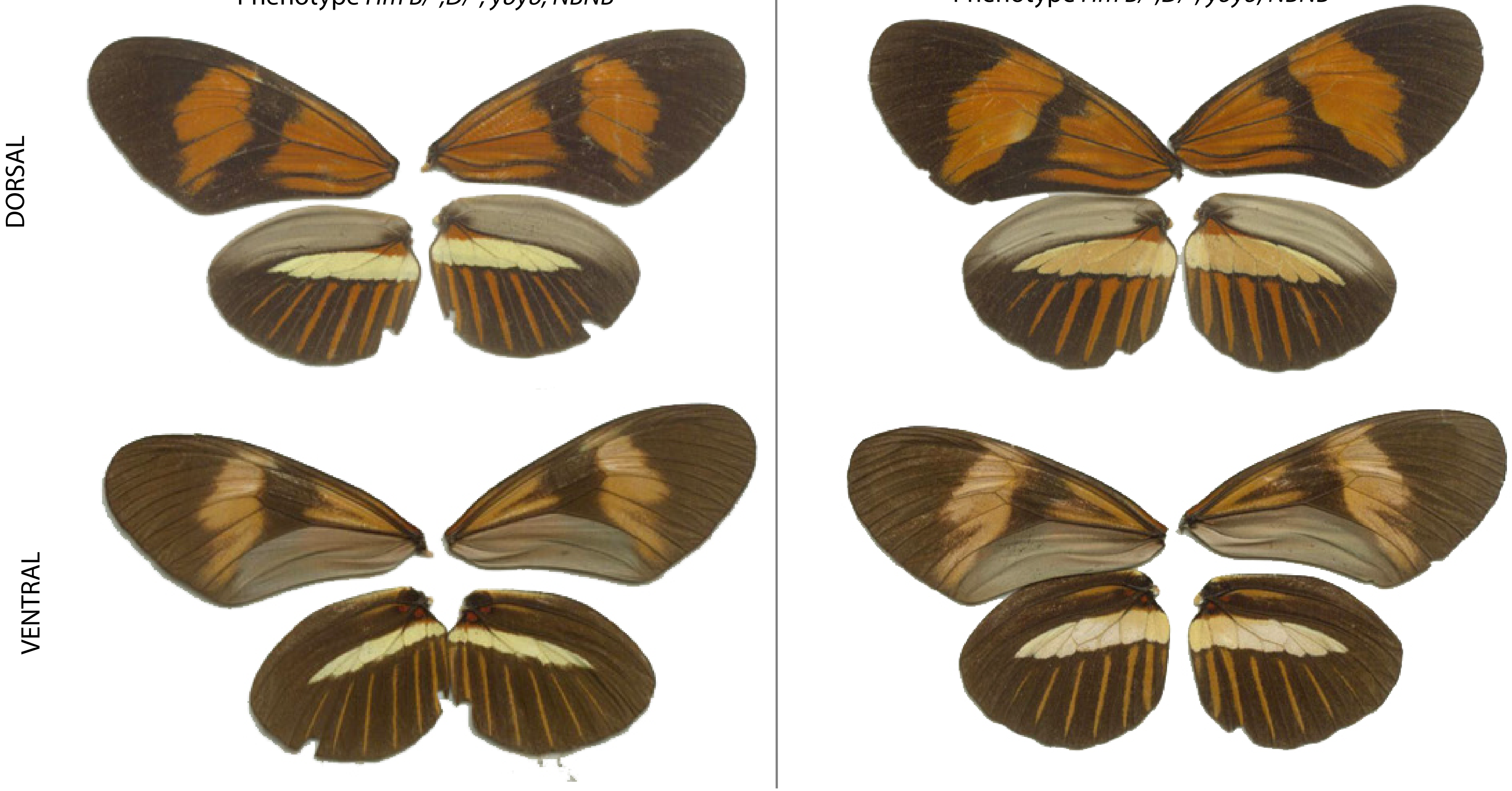

Supplement: Figure S4 — Phenotypes of two hybrid individuals sampled in the Peruvian hybrid zone. Both individuals are phenotypically most similar to H. m. amaryllis, but with expression of the dominant HmD allele from the Amazonian race controlling the red hindwing rays and basal red patch on the forewing. (1.62 MB PDF) [file pgen.1000794.s004.pdf]
