# Supplementary material for: Genomic Hotspots for Adaptation: The Population Genetics of Müllerian Mimicry in the Heliconius melpomene Clade
Source: PLoS Genet. 2010 Feb 5;6(2):e1000794. doi: 10.1371/journal.pgen.1000794 (PMC2816687; doi:10.1371/journal.pgen.1000794)
Supplement: Table S1 — Annotation of genes in the HmB region. (0.12 MB DOC) [file pgen.1000794.s005.doc]

**Table S1: Annotation of genes in the *HmB* region**

| Gene Number | GenBank Accession Number | Predicted similarity | BLAST Best Hit | e value | Predicted Function | Conserved Protein Domains | Present in BAC clone | Number of 454 transcripts | |
| --- | --- | --- | --- | --- | --- | --- | --- | --- | --- |
|  |  |  |  |  |  |  |  | *H.m. cythera* | *H. m. aglaope* |
| HM01000 | CAY54135  CAY54136  CAY54160  GQ452003 | Mad, a MAX dimerization protein | *Apis mellifera*, NP_001035923 | 3e-42 | Transcriptional repression, cell differentiation | - | bHM7G5 bHM22C5 bHM27I5 | 0 | 0 |
| HM01004 | CAY54137 | DALR anticodon binding domain | *Aedes aegypti*, XP_001651586 | 2e-52 | This all alpha helical domain is the anticodon binding domain in Arginyl and glycyl tRNA synthetase. | DALP_1 (4e-11) | bHM27I5 | 23 | 10 |
| HM01006 | CAY54138 | similar to CG7872 | *Tribolium castaneum*, XP_971385 | 1e-125 | Heat Shock protein binding. [DnaJ domain: Protein translation, folding, unfolding, translocation, and degradation] | DnaJ (1e-11) | bHM27I5 | 28 | 10 |
| HM01007 | CAY54139 | - |  |  | - | - | bHM27I5 | 2 | 0 |
| HM01009 | CAY54140 | Similar to *bves* FBgn0031150 | *Aedes aegypti*, XP_001658615 | 9e-30 | unknown function | Popeye (2e-04) | bHM27I5 | 0 | 0 |
| HM01023 | CAY54161 | - |  |  | - | - | bHM28L23 | 0 | 2 |
| HM01022 | CAY54162 | hypothetical protein | *Canis familiaris*, XP_854011 | 3e-09 |  | - | bHM28L23 | 7 | 2 |
| HM01021 | CAY54163 | phosphodiesterase 10A | *Tribolium castaneum*, XP_974266 | 2e-85 | Regulating intracellular concentration of cyclic nucleotides | GAF(5e-11) PDEase_I (5e-11) | bHM28L23 | 3 | 0 |
| HM01020 | CAY54164 | Sorting nexin | *Apis mellifera*, XP_392658 | 2e-68 | Protein targeting and transport, cell signaling | PX (6e-15) | bHM28L23 | 4 | 23 |
| HM01019 | CAY54165 | step ii splicing factor slu7 | *Tribolium castaneum*, XP_974637 | 6e-177 | nuclear mRNA splicing, via spliceosome; mitotic spindle organization and biogenesis | - | bHM28L23 | 79 | 42 |
| HM01018 | CAY54166 | kinesin-like protein | *Bombyx mori*, ABK92271 | 4e-169 | Intracellular transport and in cell division. | KISc_KIF23_like (3e-66) | bHM28L23 | 82 | 14 |
| HM01017 | CAY54167 | Probable G-protein coupled receptor 125 | *Tribolium castaneum*, XP_972198 | 1e-168 | protein binding; G-protein coupled receptor activity | LRRCT (0.006) | bHM28L23 | 2 | 1 |
| HM01014 | CAY54168 CAY54141 | epoxide hydrolase related protein | *Acyrthosiphon pisum*, XP_001943190 | 1e-14 | hydrolase or acyltransferase | MhpC (3e-05) | bHM28L23 bHM19L14 | 0 | 2 |
| HM01012 | CAY54169 | - |  |  | - | - | bHM28L23 | 2 | 0 |
| HM01028 | CAY54142 CAY54143 | Six/sine homebox transcription factors | *Culex quinquefasciatus*, XP_001868701 | 9e-93 | - | HOX (6e-08) | bHM19L14 bHM21P16 | 0 | 0 |
| HM01044 | CAY54144 | INC: integrator complex subunit 7 isoform 1 | *Apis mellifera*, XP_396796 | 9e-106 | binding | - | bHM21P16 | 0 | 0 |
| HM01043 | CAY54145 | LRR1: Leucine-rich transmembrane protein | *Tribolium castaneum*, XP_975147 | 1e-19 | - | - | bHM21P16 | 0 | 0 |
| HM01042 | CAY54146 | LRR2: Leucine-rich transmembrane protein | *Culex quinquefasciatus*, XP_001863138 | 9e-28 | protein-protein interactions | LRR_R1 (0.005) | bHM21P16 | 1 | 0 |
| HM01041 | CAY54147 | - | - | - | - | - | bHM21P16 | 21 | 2 |
| HM01040 | CAY54148 | - | - | - | - | - | bHM21P16 | 3 | 0 |
| HM01039 | CAY54149 | Strabismus/Van gogh | *Tribolium castaneum*, XP_975167 | 6e-156 | predicted to be potent tumor suppressor gene candidates | Strabismus (6e-128) | bHM21P16 | 1 | 0 |
| HM01038 | CAY54150 | Monocarboxylate transporter 14 | *Monodelphis domestica*, XP_001364940 | 2e-08 | secondary active monocarboxylate transmembrane transporter activity | - | bHM21P16 | 0 | 0 |
| HM01037 | CAY54151 | SCY1-like 2 | *Tribolium castaneum*, XP_969159 | 0.0 | protein kinase activity; ATP binding | S_TKc (6e-19) | bHM21P16 | 26 | 15 |
| HM01036 | CAY54152 CAY54170 | TM2 domain-containing protein | *Tribolium castaneum*, XP_971892 | 2e-57 | TM2 domain is composed of a pair of transmembrane alpha helices connected by a short linker. The function of this domain is unknown. | TM2 (1e-05) | bHM21P16 bHM28F19 | 42 | 12 |
| HM01035 | CAY54153 CAY54171 | 40S ribosomal protein S13 | *Spodoptera frugiperda*, Q962R6 | 1e-75 | structural constituent of ribosome | Ribosomal_s13 (4e-17) | bHM21P16 bHM28F19 | 53 | 27 |
| HM01034 | CAY54154 CAY54172 | NADH:ubiquinone dehydrogenase | *Drosophila willistoni*, XP_002062779 | 7e-17 | NADH dehydrogenase activity. Mitochondrial electron transport, NADH to ubiquinone | NDUF_B4 (2e-04) | bHM21P16 bHM28F19 | 30 | 8 |
| HM01033 | CAY54155 CAY54173 | Trafficking protein particle complex 5 | *Nasonia vitripennis*, XP_001599248 | 3e-75 | targeting and/or fusion of ER-to-Golgi transport vesicles | TRAPP_Bet3 (5e-39) | bHM21P16 bHM28F19 | 39 | 8 |
| HM01032 | CAY54156 CAY54174 | - | - | - | - | - | bHM21P16 bHM28F19 | 8 | 2 |
| HM01031 | CAY54157 CAY54175 | Ras-related protein Rab-39B | *Apis mellifera*, XP_623117 | 3e-107 | small GTPase mediated signal transduction; protein transport. | Rab39 (8e-87) | bHM21P16 bHM28F19 | 4 | 3 |
| HM01030 | CAY54159 CAY54177 | - | - | - | - | - | bHM21P16 bHM28F19 | 6 | 7 |
| HM01029 | CAY54158 CAY54176 | THAP domain-containing protein | *Salmo salar*, ACI68488 | 4e-08 | - | THAP (2e-09) | bHM21P16 bHM28F19 | 42 | 7 |
