# Supplementary material for: Genomic Hotspots for Adaptation: The Population Genetics of Müllerian Mimicry in the Heliconius melpomene Clade
Source: PLoS Genet. 2010 Feb 5;6(2):e1000794. doi: 10.1371/journal.pgen.1000794 (PMC2816687; doi:10.1371/journal.pgen.1000794)
Supplement: Table S2 — Gene sequencing and quantitative PCR primers. (0.40 MB DOC) [file pgen.1000794.s006.doc]

**Table S2: Gene sequencing and quantitative PCR primers**

|  | Locus tag | HE gene number | HM gene number | Predicted Gene | Primer Name | Primer Sequence | GenBank Accession | size bp (exon/intron) | Tm | INTRON? | BAC clone | Linkage Group |
| --- | --- | --- | --- | --- | --- | --- | --- | --- | --- | --- | --- | --- |
| HmB locus primers for gene sequencing |  |  |  |  |  |  |  |  |  |  |  |  |
|  | *Dna-J* | HEC0300004 | HM01006 | Similar to CG7872 | Sim_CG7872_F | GTGCTCGGTGTCACAAGAGA | CU467807 | 624/0 | 58 | No | AEHM-27I5 | LG18 |
|  |  |  |  | (Dna-J domain) | Sim_CG7872_R | CTTCCAAAGCCATCTTGCAT |  |  |  |  |  |  |
|  |  |  |  |  |  |  |  |  |  |  |  |  |
|  | *Slu7* | HEC0300006 | HM01019 | Step ii splicing factor slu7 | slu7_F | GCGTGTGGCAGTATCTCAAA | CU467808 | 256/192 | 58 | Yes | AEHM-28L23 | LG18 |
|  |  |  |  |  | slu7_R | ATCCTCTTGTGGTCGCTGAT |  |  |  |  |  |  |
|  |  |  |  |  |  |  |  |  |  |  |  |  |
|  | *kinesin* | HEC0300007 | HM01018 | Kinesin-like | Kinesin_F | TTGAACCTAGGCCACCTGTAA | CU467808 | 487/72 | 58 | Yes | AEHM-28L23 | LG18 |
|  |  |  |  |  | Kinesin_R | TCTGGACTTTGAGCAACATCA |  |  |  |  |  |  |
|  |  |  |  |  |  |  |  |  |  |  |  |  |
|  | *GPCR* | HEC0300008 | HM01017 | G protein coupled receptor | GPCR-F | CCAAAATGTGCATTTCCTGA | CU467808 | 672/0 | 58 | No | AEHM-28L23 | LG18 |
|  |  |  |  |  | GPCR-R | CATGCCAAGTCGTTCTGTTG |  |  |  |  |  |  |
|  |  |  |  |  |  |  |  |  |  |  |  |  |
|  | *LRR(2)* | - | HM01042 | Leucine rich repeat (2) | LRR_2_F | CGACGAAATGTTGTTTCACG | CU681835 | 973/0 | 56 | No | AEHM-21P16 | LG18 |
|  |  |  |  |  | LRR_2_R | CCTCGATCCCTTCAACGATA |  |  |  |  |  |  |
|  |  |  |  |  |  |  |  |  |  |  |  |  |
|  | *VanGogh* | HEC0300010 | HM01038 | Strabismus/Van Gogh | Str_F | ATGGCACCATTCCAAACAAG | CU681835 | 744/0 | 58 | No | AEHM-21P16 | LG18 |
|  |  |  |  |  | Str_R | TGACCTGCTTTCACCATCTG |  |  |  |  |  |  |
|  |  |  |  |  |  |  |  |  |  |  |  |  |
|  | *SCY1* | - | HM01037 | SCY1-like 2 | SCY1_F | ATGGTCGAAAACGGACAGAG | CU681835 | 732/0 | 56 | No | AEHM-21P16 | LG18 |
|  |  |  |  |  | SCY1_R | GGGTCAGGTCGTAATTCAGG |  |  |  |  |  |  |
|  |  |  |  |  |  |  |  |  |  |  |  |  |
|  | *MRSP* | - | - | Methionine Rich Storage Protein | MRSP_F1 | GAACACGCTGTGCTTTTCAA | EU711404 | 639/0 | 59 | No | N/A | LG18 |
|  |  |  |  |  | MRSP_R1 | AATGGTTGGCAGAACCAGAC |  |  |  |  |  |  |
|  |  |  |  |  |  |  |  |  |  |  |  |  |
| HmYb locus primers for gene sequencing |  |  |  |  |  |  |  |  |  |  |  |  |
|  | *Fork* | HEC0500001 | - | Forkhead-box | ForkBox_Int_F | CTGCAGATTGGGCTGATTTT |  | 552/88 | 55 | Yes | AEHM-41C10 | LG15 |
|  |  |  |  |  | ForkBox_Int_R | CATTCGATTTCAACGGTGTG |  |  |  |  |  |  |
|  |  |  |  |  |  |  |  |  |  |  |  |  |
|  | *Treh1B* | HEC050003 | HM00004 | Trehalase-1B | Trehalase_1_F4 | TATGTTCCGCTTTATCCTGT |  | 666/0 | 60 | No | AEHM-7G12 | LG15 |
|  |  |  |  |  | Trehalase_1_RTR3 | CGCTAATAAAGGTGGTTGAC |  |  |  |  |  |  |
|  |  |  |  |  |  |  |  |  |  |  |  |  |
|  | *Treh1A* | - | HM00006 | Trehalase-1A | Trehalase-F907 | AGCCCTGAACGTAAGCAAGA |  | 750/0 | 56 | No | AEHM-7G12 | LG15 |
|  |  |  |  |  | Trehalase-R1736 | ACATGAGCACCCTCAACTCC |  |  |  |  |  |  |
|  |  |  |  |  |  |  |  |  |  |  |  |  |
|  | *B9* | HEC050004 | HM00007 | B9 protein | CG14870-F8 | TGCGAGAATCTGGAGTAACAAA |  | 444/585 | 56 | Yes | AEHM-7G12 | LG15 |
|  |  |  |  |  | CG14870-R546 | GGTCTACCAGCTCTGGATGC |  |  |  |  |  |  |
|  |  |  |  |  |  |  |  |  |  |  |  |  |
|  | *HM00008* | - | HM00008 | - | gene24_ex4_F76006 | ACCAGAAAAACCACCGACAC |  | 586/0 | 55 | No | AEHM-7G12 | LG15 |
|  |  |  |  |  | gene24_ex4_R76589 | CAGATTTTCGTCGCTTCCTC |  |  |  |  |  |  |
|  |  |  |  |  |  |  |  |  |  |  |  |  |
|  | *WD40 exon 4* | HEC0500005 | HM00010-exon 4 | WD40 repeat domain 85 | gene26_F83921 | GACTCGTCGGGTGCAGTAAC |  | 313/592 | 55 | Yes | AEHM-7G12 | LG15 |
|  |  |  |  |  | gene26_R82997 | TCTCGGTGATGCAACGTTTA |  |  |  |  |  |  |
|  |  |  |  |  |  |  |  |  |  |  |  |  |
|  | *WD40 exon 5* | HEC0500005 | HM00010-exon 5 | WD40 repeat domain 85 | WD_F2 | TTTTACAGTTATGACGAAAAAGTCC |  | 1046 | 60 | No | AEHM-7G12 | LG15 |
|  |  |  |  |  | WD_R2 | GGCTGCATCAATTGAAAAGA |  |  |  |  |  |  |
|  |  |  |  |  |  |  |  |  |  |  |  |  |
|  | *Unkempt* | HEC0500006 | HM00013 | unkempt | unkempt_in3_F228 | TCCAGATGGAACTGATGGTG |  | 167/471 | 57 | Yes | AEHM-11J7 | LG15 |
|  |  |  |  |  | unkempt_in3_R394 | GGCAGGCATACCCTTGTCTA |  |  |  |  |  |  |
|  |  |  |  |  |  |  |  |  |  |  |  |  |
|  | *recQ* | HEC0500007 | HM00017 | RecQ Helicase | Helicase_F125 | GCTGGCCTATCCAAATGAAA |  | 945/0 | 56 | No | AEHM-11J7 | LG15 |
|  |  |  |  |  | Helicase_R1170 | ACAGCGACTGCTACAGCTCA |  |  |  |  |  |  |
|  |  |  |  |  |  |  |  |  |  |  |  |  |
|  |  | - | HM00019 | BmSuc2 | Invertase_F415 | GGAGATGGGGTGTTCTTTCA |  | 611/0 | 56 | No | AEHM-11J7 | LG15 |
|  |  |  |  |  | Invertase_R1021 | TGGGAGACATGAGGAGAACC |  |  |  |  |  |  |
|  |  |  |  |  |  |  |  |  |  |  |  |  |
|  |  | - | HM00021-exon 6 | - | hm21_ex5_F1 | AGATTCATAAACCACCAGATATTGA |  | 764/0 | 58 | No | AEHM-11J7 AEHM-29B7 | LG15 |
|  |  |  |  |  | hm21_ex5_R1 | TTAAAATATACTATCCAGCAAAGATCC | |  |  |  |  |  |
|  |  |  |  |  |  |  |  |  |  |  |  |  |
|  |  | - | HM00021-exon 3-4 | - | Gene21_in2-3_R564 | ACGACGCAGGTATTGTGTCA |  | 240/446 | 56 | Yes | AEHM-11J7 AEHM-29B7 | LG15 |
|  |  |  |  |  | Gene21_in2-3_F172 | CCTCCTGAATGTGGCAAATC |  |  |  |  |  |  |
|  |  |  |  |  |  |  |  |  |  |  |  |  |
|  |  | - | HM00023-exon 3 | ATP binding protein | ATPK_F2 | AGACGAAATTGGCAAAATGG |  | 161/0 | 55 | No | AEHM-11J7 AEHM-29B7 | LG15 |
|  |  |  |  |  | ATPK_R2 | ACCATCCAAATTTTTGTTTTGTTA |  |  |  |  |  |  |
|  |  |  |  |  |  |  |  |  |  |  |  |  |
|  | LRR exon 3 | HEC0500009 | HM00024-exon 3 | Leucine rich repeat | LLR_F2 | TTTAGTTGCAGATGTAGATAACCAAT | | 401/0 | 57 | No | AEHM-11J7 AEHM-29B7 | LG15 |
|  |  |  |  |  | LLR_R2 | TTCTGAATTTCCATTTGCCTA |  |  |  |  |  |  |
|  |  |  |  |  |  |  |  |  |  |  |  |  |
|  | LRR exon 2 | HEC0500009 | HM00024-exon 2 | Leucine rich repeat | LRR_F4 | GTAGAAAAAGAGAGACCTGAAAGTG |  | 195/711 | 55 | Yes | AEHM-11J7 AEHM-29B7 | LG15 |
|  |  |  |  |  | LRR_R6 | ATAAAGCCATGGTGACTGTGTA |  |  |  |  |  |  |
|  |  |  |  |  |  |  |  |  |  |  |  |  |
|  | LRR exon 1 | HEC0500009 | HM00024-exon 1 | Leucine rich repeat | Gene22bTrib-ex1_Ro | GATGTCCCCAAACAAATGGA |  | 540/0 | 53 | No | AEHM-11J7 AEHM-29B7 | LG15 |
|  |  |  |  |  | Gene22bTrib-ex1_Fo | TGGAGGAAAATGAAAACAAACC |  |  |  |  |  |  |
|  |  |  |  |  |  |  |  |  |  |  |  |  |
|  |  | - | HM00023-exon 10-11 | ATP binding protein | 11J7_gene25_f1 | TTGACTGTTGTGGTATCAGA |  | 323/400 | 55 | Yes | AEHM-11J7 AEHM-29B7 | LG15 |
|  |  |  |  |  | 11J7_gene25_r1 | AGAAGTTTAATTGGTTCCGT |  |  |  |  |  |  |
|  |  |  |  |  |  |  |  |  |  |  |  |  |
|  |  | - | HM00023-exon 11-12 | ATP binding protein | 11J7_gene25_f2 | GACTCCCAGACAAATATACT |  | 230/425 | 55 | Yes | AEHM-11J7 AEHM-29B7 | LG15 |
|  |  |  |  |  | 11J7_gene25_r2 | TTATCTATAGAACAAATTGC |  |  |  |  |  |  |
|  |  |  |  |  |  |  |  |  |  |  |  |  |
|  |  |  | - | Intergenic | Gene24Trib-ex2_F11 | TTTATCAAACACGCCCGAAT |  | 302 | 55 | - | AEHM-11J7 AEHM-29B7 | LG15 |
|  |  |  |  |  | Gene24Trib-ex2_R311 | CAGACATAGGCCTCCTCCAA |  |  |  |  |  |  |
|  |  |  |  |  |  |  |  |  |  |  |  |  |
|  |  |  | - | Intergenic | Banksy_For | GACGGATAGCGCACATACAAAACA |  | 510 | 55 | - | AEHM-11J7 AEHM-29B7 | LG15 |
|  |  |  |  |  | Banksy_Rev | CGGCCCAATAGAAGCGATAGGA |  |  |  |  |  |  |
|  |  |  |  |  |  |  |  |  |  |  |  |  |
|  |  |  | - | Intergenic | Ricky_For | TTCCCGCAATATCTCAATCTCAT |  | 527 | 55 | - | AEHM-29B7 | LG15 |
|  |  |  |  |  | Ricky_Rev | CATCGCCGTCGCATAAACAATA |  |  |  |  |  |  |
|  |  |  |  |  |  |  |  |  |  |  |  |  |
|  |  |  | - | Intergenic | Howard3_For | GCCGGGGCAACGACCTC |  | 586 | 55 | - | AEHM-29B7 | LG15 |
|  |  |  |  |  | Howard3_Rev | CCGGCGGGAAGATGTGTAAAA |  |  |  |  |  |  |
|  |  |  |  |  |  |  |  |  |  |  |  |  |
| Genes unlinked to colour pattern |  |  |  |  |  |  |  |  |  |  |  |  |
|  |  |  | - | CDP | cdpF | AACAAAATGGGAAAAACATC | GQ452006 | 802/0 | 58 | No | N/A | N/A |
|  |  |  |  |  | cdpR | TCCTTGTATGGGGGATTGAT |  |  |  |  |  |  |
|  |  |  |  |  |  |  |  |  |  |  |  |  |
|  |  |  | - | DEAD5 | DEAD5-F | GAGATAACAGTTAGTGGCAAT | GQ452007 | 715/0 | 55 | No | N/A | N/A |
|  |  |  |  |  | DEAD5-R | TCCTTCTCATGTTCTTCACA |  |  |  |  |  |  |
|  |  |  |  |  |  |  |  |  |  |  |  |  |
|  | Caspase |  | - | Caspase-1 | Caspase_F | AATGCCTGTGGAAAGAAACG | EF211965 | 338/0 | 58 | No | N/A | LG20 |
|  |  |  |  |  | Caspase_R | ACCAGAGGGTGTCAGGCTTA |  |  |  |  |  |  |
|  |  |  |  |  |  |  |  |  |  |  |  |  |
|  | rpl10A |  | - | Ribosomal Protein - gene L10A | RpL10A_F | ACAGTGGAACTCCAAATCGG | CO729740  GQ452004 | 182/412 | 58 | Yes | N/A | LG11 |
|  |  |  |  |  | RpL10A_R | TTTAGAGCCTCGGCATCCAT |  |  |  |  |  |  |
|  |  |  |  |  |  |  |  |  |  |  |  |  |
|  | Ubx |  | - | Ultrabithorax | Ubx_F | TGCAGGAGAGCAGCAATATG | GQ452005 | 271/0 | 58 | No | N/A | LG3 |
|  |  |  |  |  | Ubx_R | TATGGCCATCCAAGGGTAGA |  |  |  |  |  |  |
|  |  |  |  |  |  |  |  |  |  |  |  |  |
| Primers for Quantitative PCR |  |  |  |  |  |  |  |  |  |  |  |  |
|  | Mad |  | HM01000 | Mad | Mad_qPCR_F | CGAGTTGGAAAAGAACAGA | CU467807 CU462858  GQ452003 | 207 cDNA | 60 | Yes | AEHM-7G5 AEHM-27I5 | LG18 |
|  |  |  |  |  | Mad_qPCR_R | CGTCGTAGGTAGCGTTGT |  |  |  |  |  |  |
|  |  |  |  |  |  |  |  |  |  |  |  |  |
|  | Kinesin-like |  | HM01018 | Kinesin-like | Kinesin_qPCR_F | AGATATAGATGAAGATACAAAGAAA | CU467808 | 236 cDNA | 58 | Yes | AEHM-28L23 | LG18 |
|  |  |  |  |  | Kinesin_qPCR_R | TGAATAACTTTCGCCTAGT |  |  |  |  |  |  |
|  |  |  |  |  |  |  |  |  |  |  |  |  |
|  | GPCR |  | HM01017 | G-protein coupled receptor | GPCR_qPCR_F | TAAGAGGGCACAGAGCTAAG | CU467808 | 158 cDNA | 60 | Yes | AEHM-28L23 | LG18 |
|  |  |  |  |  | GPCR_qPCR_R | CACCGTCGCTATTATAGGTT |  |  |  |  |  |  |
|  |  |  |  |  |  |  |  |  |  |  |  |  |
|  | slu7 |  | HM01019 | slu7 | slu7_qPCR_F | GAAAACTGTGGAGCCATGA | CU467808 | 92 bp cDNA | 55.4 | Yes | AEHM-28L23 | LG18 |
|  |  |  |  |  | slu7_qPCR_R | GAAGCTATGCCT*GCACTT*G |  |  |  |  |  |  |
|  |  |  |  |  |  |  |  |  |  |  |  |  |
|  | EF1-a |  | - | EF1-a | EF1-a_qPCR_F | GCTGACGGTAAATGCCTCAT | AY747994  GQ452009 | 181 cDNA | 60 | No | N/A | LG10 |
|  |  |  |  |  | EF1-a_qPCR_R | CAGGAGCGAACAC*AACAAT*G |  |  |  |  |  |  |
|  |  |  |  |  |  |  |  |  |  |  |  |  |
|  | RpS3A |  | - | RpS3A | RpS3A_qPCR_F | TAGATCCCTTCACAAGGAAA | GQ452008 | 169 cDNA | 58 | Yes | N/A | N/A |
|  |  |  |  |  | RpS3A_qPCR_R | GTATCAGCTTGGAGATCAGC |  |  |  |  |  |  |
