# Supplementary material for: Genomic Hotspots for Adaptation: The Population Genetics of Müllerian Mimicry in the Heliconius melpomene Clade
Source: PLoS Genet. 2010 Feb 5;6(2):e1000794. doi: 10.1371/journal.pgen.1000794 (PMC2816687; doi:10.1371/journal.pgen.1000794)
Supplement: Table S3 — Summary of gene regions sequenced for population genetic analysis. (0.12 MB DOC) [file pgen.1000794.s007.doc]

***Table S3: Summary of gene regions sequenced for population genetic analysis***

| *Gene name* | *Region* | *Start (bp)* | *length* | *H. m. ros.* | *H. m. mel.* | *H. m. mel.* | *H. m agl.* | *H. m ama.* | *H. cydno* | *H. pach.* |
| --- | --- | --- | --- | --- | --- | --- | --- | --- | --- | --- |
|  |  |  |  | Panama | Venezuela | Panama | Peru | Peru |  |  |
|  |  |  |  | Number of individuals | | | | | | |
| GDH | Yb | -14457 | 194 | - | - | - | - | - | 6 | 9 |
| Forkhead-box | Yb | -9536 | 624 | 6 | 5 | - | - | - | - | - |
| HM00004 | Yb | 35991 | 586 | 7 | 7 | - | 5 | 5 | - | - |
| HM00006 | Yb | 40777 | 694 | 13 | 5 | - | 6 | 8 | 7 | 6 |
| HM00007 | Yb | 63064 | 497 | 19 | 8 | - | - | - | 5 | 7 |
| HM00008 | Yb | 76578 | 436 | 8 | 8 | - | 30 | 31 | - | - |
| HM00010 exon 4 | Yb | 83038 | 402 | 12 | 8 | 8 | 20 | 26 | - | - |
| HM00010 exon 5 | Yb | 83782 | 562 | 14 | 6 | 6 | 11 | 6 | - | - |
| HM00013 | Yb | 122806 | 598 | 6 | 5 | - | 6 | 5 | - | - |
| HM00017 | Yb | 137287 | 919 | 7 | 7 | - | - | - | 5 | 6 |
| HM00019 | Yb | 150432 | 557 | 10 | 7 | - | - | - |  |  |
| HM00021 exon 6 | Yb | 168406 | 659 | 13 | 5 | 7 | - | - | 5 | 5 |
| HM00021 exon 3-4 | Yb | 169658 | 658 | 5 | 6 | - | - | - | - | - |
| HM00022 | Yb | 174282 | 121 | - | - | - | - | - | 5 | 8 |
| HM00024 exon 3 | Yb | 180098 | 334 | 17 | 8 | 7 | 30 | 31 | 6 | 8 |
| HM00024 exon 2 | Yb | 180727 | 700 | 16 | 7 | 8 | - | - | 8 | 10 |
| HM00024 exon 1 | Yb | 181482 | 473 | 11 | 7 | - | 9 | 10 | 19 | 12 |
| HM00023 exon 10-11 | Yb | 183036 | 683 | 12 | 7 | - | - | - | - | - |
| HM00023 exon 11-12 | Yb | 183508 | 447 | 12 | 5 | 8 | - | - | - | - |
| Non-coding | Yb | 183553 | 532 | - | - | - | - | - | 33 | 22 |
| Non-coding | Yb | 184965 | 255 | 8 | 5 | - | - | - | - | - |
| Non-coding | Yb | 188104 | 464 | 14 | - | 7 | - | - | - | - |
| Non-coding | Yb | 188182 | 290 | - | - | - | - | - | 12 | 12 |
| Non-coding | Yb | 218906 | 183 | - | - | - | - | - | 12 | 9 |
| Non-coding | Yb | 218975 | 482 | 16 | 7 | 8 | - | - | - | - |
| Non-coding | Yb | 258132 | 548 | 16 | 6 | 6 | - | - | - | - |
| Caspase | Unlinked |  | 299 | 12 | 6 | 8 | 30 | 32 | - | - |
| CDP | Unlinked |  | 762 | 14 | 6 | 8 | - | - | - | - |
| DEAD5 | Unlinked |  | 674 | 13 | 8 | 7 | - | - | - | - |
| RPL10A | Unlinked |  | 557 | 16 | 8 | 7 | 27 | 27 | - | - |
| UBX | Unlinked |  | 230 | 16 | 8 | 8 | 30 | 31 | - | - |
| CG7872 | B | 266420 | 624 | - | - | - | 30 | 30 | - | - |
| Slu7 | B | 345169 | 448 | - | - | - | 30 | 30 | - | - |
| Kinesin | B | 359486 | 559 | - | - | - | 30 | 30 | - | - |
| GPCR | B | 362828 | 672 | - | - | - | 30 | 30 | - | - |
| LRR-2 | B | 573803 | 973 | - | - | - | 30 | 30 | - | - |
| Strabismus | B | 585117 | 744 | - | - | - | 30 | 30 | - | - |
| SCY1 | B | 598036 | 732 | - | - | - | 30 | 30 | - | - |
| MRSP | B | 1000000 | 639 | - | - | - | 30 | 30 | - | - |
