# Supplementary material for: Genomic Hotspots for Adaptation: The Population Genetics of Müllerian Mimicry in the Heliconius melpomene Clade
Source: PLoS Genet. 2010 Feb 5;6(2):e1000794. doi: 10.1371/journal.pgen.1000794 (PMC2816687; doi:10.1371/journal.pgen.1000794)
Supplement: Table S6 — Estimates of Tajima's D and nucleotide diversity estimates for each locus by population. (0.28 MB DOC) [file pgen.1000794.s010.doc]

Table S6: Estimates of Tajima’s D and nucleotide diversity (π) estimates for each locus by population.

| Region name | Position | Population | Tajima's D |  | π |
| --- | --- | --- | --- | --- | --- |
| *HmYb* locus |  |  |  |  |  |
| *GDH* | -14457 | cydno | -0.279 |  | 0.0079 |
|  |  | pachinus | 1.537 |  | 0.0049 |
| *F-box* | -9536 | rosina | 1.220 |  | 0.0102 |
|  |  | melpomene(Venezuela) | 0.718 |  | 0.0105 |
| HM00004 | 35991 | rosina | -0.589 |  | 0.0041 |
|  |  | melpomene(Venezuela) | 0.123 |  | 0.0066 |
|  |  | aglaope | 0.297 |  | 0.0096 |
|  |  | amaryllis | 1.310 |  | 0.0071 |
| HM00006 | 40777 | rosina | 0.438 |  | 0.0177 |
|  |  | melpomene(Venezuela) | 1.051 |  | 0.0144 |
|  |  | aglaope | 0.091 |  | 0.0166 |
|  |  | amaryllis | 0.940 |  | 0.0181 |
|  |  | cydno | -0.128 |  | 0.0038 |
|  |  | pachinus | -0.299 |  | 0.0038 |
| HM00007 | 63064 | rosina | -1.024 |  | 0.0103 |
|  |  | melpomene(Venezuela) | 1.854 |  | 0.0205 |
|  |  | cydno | 0.143 |  | 0.0114 |
|  |  | pachinus | -1.481 |  | 0.0022 |
| HM00008 | 76578 | rosina | 0.184 |  | 0.0023 |
|  |  | melpomene(Venezuela) | 0.735 |  | 0.0026 |
|  |  | aglaope | -1.000 |  | 0.0067 |
|  |  | amaryllis | -1.817 | * | 0.0021 |
| HM00010 *(ex4)* | 83038 | rosina | -1.195 |  | 0.0034 |
|  |  | melpomene(Venezuela) | -0.555 |  | 0.0025 |
|  |  | melpomene(Panama) | -1.124 |  | 0.0077 |
|  |  | aglaope | -1.250 |  | 0.0084 |
|  |  | amaryllis | -0.790 |  | 0.0063 |
| HM00010 *(ex5)* | 83782 | rosina | -0.340 |  | 0.0030 |
|  |  | melpomene(Venezuela) | -0.718 |  | 0.0026 |
|  |  | melpomene(Panama) | 0.255 |  | 0.0053 |
|  |  | aglaope | -0.025 |  | 0.0054 |
|  |  | amaryllis | 1.072 |  | 0.0050 |
| HM00013 | 122806 | rosina | -0.382 |  | 0.0045 |
|  |  | melpomene(Venezuela) | -2.051 | ** | 0.0554 |
|  |  | aglaope | 0.417 |  | 0.0142 |
|  |  | amaryllis | 0.169 |  | 0.1141 |
| HM00017 | 137287 | rosina | -0.595 |  | 0.0020 |
|  |  | melpomene(Venezuela) | -0.860 |  | 0.0046 |
|  |  | cydno | -0.747 |  | 0.0056 |
|  |  | pachinus | -0.952 |  | 0.0028 |
| HM00019 | 150432 | rosina | 0.234 |  | 0.0107 |
|  |  | melpomene(Venezuela) | -1.134 |  | 0.0053 |
| HM00021 *(ex6)* | 168406 | rosina | 0.766 |  | 0.0051 |
|  |  | melpomene(Venezuela) | 0.049 |  | 0.0071 |
|  |  | melpomene(Panama) | -0.689 |  | 0.0080 |
|  |  | cydno | -0.234 |  | 0.0073 |
|  |  | pachinus | -1.796 | * | 0.0022 |
| HM00021 *(ex3-4)* | 169658 | rosina | -0.076 |  | 0.0292 |
|  |  | melpomene(Venezuela) | -0.108 |  | 0.0080 |
| HM00022 | 174282 | cydno | 0.305 |  | 0.0312 |
|  |  | pachinus | -0.978 |  | 0.0107 |
| HM00024 *(ex3)* | 180098 | rosina | -1.971 | * | 0.0030 |
|  |  | melpomene(Venezuela) | -0.253 |  | 0.0068 |
|  |  | melpomene(Panama) | 0.430 |  | 0.0085 |
|  |  | aglaope | -0.651 |  | 0.0088 |
|  |  | amaryllis | -0.614 |  | 0.0056 |
|  |  | cydno | -0.894 |  | 0.0135 |
|  |  | pachinus | -0.997 |  | 0.0067 |
| HM00024 *(ex2)* | 180727 | rosina | -0.662 |  | 0.0088 |
|  |  | melpomene(Venezuela) | -0.652 |  | 0.0082 |
|  |  | melpomene(Panama) | -0.280 |  | 0.0118 |
|  |  | cydno | -0.883 |  | 0.0103 |
|  |  | pachinus | -0.143 |  | 0.0067 |
| HM00024 *(ex1)* | 181482 | rosina | -0.525 |  | 0.0069 |
|  |  | melpomene(Venezuela) | -0.778 |  | 0.0053 |
|  |  | aglaope | 0.326 |  | 0.0083 |
|  |  | amaryllis | 1.270 |  | 0.0089 |
|  |  | cydno | -1.771 |  | 0.0046 |
|  |  | pachinus | 0.285 |  | 0.0014 |
| *HM00023 (ex10-11)* | 183036 | rosina | 0.192 |  | 0.0079 |
|  |  | melpomene(Venezuela) | -0.750 |  | 0.0186 |
| *HM00023 (ex11-12)* | 183508 | rosina | -0.665 |  | 0.0100 |
|  |  | melpomene(Venezuela) | -1.137 |  | 0.0099 |
|  |  | melpomene(Panama) | -0.156 |  | 0.0197 |
|  |  | cydno | -1.044 |  | 0.0107 |
|  |  | pachinus | -0.059 |  | 0.0068 |
| *Gene 26* | 184965 | rosina | 1.058 |  | 0.0211 |
|  |  | melpomene(Venezuela) | 0.482 |  | 0.0245 |
| *Banksy* | 188104 | rosina | -0.030 |  | 0.0106 |
|  |  | melpomene(Panama) | 0.798 |  | 0.0162 |
|  |  | cydno | -0.254 |  | 0.0192 |
|  |  | pachinus | 1.264 |  | 0.0135 |
| *Ricky* | 218975 | rosina | -0.562 |  | 0.0084 |
|  |  | melpomene(Venezuela) | -0.745 |  | 0.0205 |
|  |  | melpomene(Panama) | 0.021 |  | 0.0090 |
|  |  | cydno | 0.049 |  | 0.0151 |
|  |  | pachinus | -0.273 |  | 0.0191 |
| *Howard* | 258132 | rosina | -0.878 |  | 0.0098 |
|  |  | melpomene(Venezuela) | 1.096 |  | 0.0169 |
|  |  | melpomene(Panama) | 0.774 |  | 0.0159 |
| Unlinked |  |  |  |  |  |
| *caspase* | *unlinked* | rosina | -0.607 |  | 0.0013 |
|  |  | melpomene(Venezuela) | 0.026 |  | 0.0089 |
|  |  | melpomene(Panama) | -1.798 | * | 0.0029 |
|  |  | aglaope | -1.036 |  | 0.0063 |
|  |  | amaryllis | 0.138 |  | 0.0068 |
| *CDP* | *unlinked* | rosina | -0.333 |  | 0.0009 |
|  |  | melpomene(Venezuela) | -0.946 |  | 0.0023 |
|  |  | melpomene(Panama) | -1.072 |  | 0.0016 |
| *DEAD5* | *unlinked* | rosina | -0.960 |  | 0.0004 |
|  |  | melpomene(Venezuela) | -1.038 |  | 0.0005 |
|  |  | melpomene(Panama) | -0.850 |  | 0.0007 |
| *RpL10a* | *unlinked* | rosina | -1.054 |  | 0.0056 |
|  |  | melpomene(Venezuela) | -1.646 |  | 0.0044 |
|  |  | melpomene(Panama) | -2.001 | * | 0.0140 |
|  |  | aglaope | 0.874 |  | 0.0255 |
|  |  | amaryllis | 1.324 |  | 0.0236 |
| *UBX* | *unlinked* | rosina | 0.650 |  | 0.0080 |
|  |  | melpomene(Venezuela) | 0.552 |  | 0.0091 |
|  |  | melpomene(Panama) | 0.521 |  | 0.0091 |
|  |  | aglaope | -1.594 |  | 0.0052 |
|  |  | amaryllis | -1.643 |  | 0.0060 |
| *HmB* locus |  |  |  |  |  |
| CG7872 | 266420 | aglaope | -0.994 |  | 0.0025 |
|  |  | amaryllis | -0.769 |  | 0.0017 |
| Slu7 | 345169 | aglaope | -0.584 |  | 0.0068 |
|  |  | amaryllis | -1.344 |  | 0.0064 |
| Kinesin | 359486 | aglaope | -1.361 |  | 0.0058 |
|  |  | amaryllis | 1.07 |  | 0.0143 |
| GPCR | 362828 | aglaope | -0.955 |  | 0.0046 |
|  |  | amaryllis | -0.798 |  | 0.0038 |
| LRR-2 | 573803 | aglaope | -1.819 | * | 0.0046 |
|  |  | amaryllis | -0.88 |  | 0.0037 |
| Strabismus | 585117 | aglaope | -2.078 | * | 0.0020 |
|  |  | amaryllis | -1.174 |  | 0.0014 |
| SCY1 | 598036 | aglaope | -1.968 | * | 0.0041 |
|  |  | amaryllis | 0.727 |  | 0.0030 |
| MRSP | 1000000 | aglaope | -1.508 |  | 0.0031 |
|  | (estimate) | amaryllis | -1.212 |  | 0.0018 |

*s indicate significance: * p<0.05, ** p<0.01. Note that Bonferroni corrections applied to all populations would make all tests non-significant.

Note that large regions of IN/DEL polymorphism were removed from the Slu7, Kinesin and RpL10a sequences prior to analysis.
