# Supplementary material for: Genomic Hotspots for Adaptation: The Population Genetics of Müllerian Mimicry in the Heliconius melpomene Clade
Source: PLoS Genet. 2010 Feb 5;6(2):e1000794. doi: 10.1371/journal.pgen.1000794 (PMC2816687; doi:10.1371/journal.pgen.1000794)
Supplement: Text S1 — Supplementary methods. (0.03 MB DOC) [file pgen.1000794.s011.doc]

**Supplementary Methods**

**Statistical methods for quantitative PCR.**

Our qPCR expression measurements are relative not absolute; therefore, gene expression values reported here are only meaningful for samples in this study. We defined *C*_t_ values as the point at which fluorescence crossed a threshold (R_Ct_) of 10 × the standard deviation of the background fluorescence and calculated amplification efficiencies (*E*) using a dilution series of locus specific PCR product (10^-3^× to 10^-9^×). The initial amount of transcript was estimated as *R_o_*=*R*_Ct_ (1+*E*)^-^*^C^*^t^. To calculate normalized values, we divided *R_o_* values for target loci by *R_o_* values of the average between *EF1-α* and *RpS3A* (for ‘between wing segments experiment) or *EF1-α* (for ‘between races’ experiment). Finally, the mean normalized value was chosen to represent a standard 1× expression, and all other sample values were adjusted in relation to this standard.

To draw statistical inferences, we use Bayesian model selection. This approach allows different models to be compared by evaluating their relative support from the observed data and model averaging can be used to make very robust estimates [1]. We used generalized linear regression models (GLMs) in combination with Bayesian Model Averaging (BMA) to model the effect of the explanatory variables “D” (i.e. Developmental Stage and Wing Segment for ‘between wing segments’ experiments, and Race, Developmental Stage and Wing Position for ‘between races’ experiment) on the independent variable “*E*” (i.e. gene expression). For both qPCR experiments we considered full models; that is a model including all possible interactions between explanatory variables. Residuals of all the performed GLMs were analysed by visual inspection and no deviations from normality were observed. No significant outliers were found using Cook’s statistic values.

The model averaging was calculated as:

Where, *P(E|D,Mk,D)* is the posterior prediction from model Mk, given the data, *P(Mk,|D)* is the posterior probability of model *M_k_*, given the data and *k* is the number of models considered [2]. That is, BMA provides an estimate of P(*E*|D) that is a weighted average of the posterior prediction from all models considered, where the weights are the posterior probabilities of each model. Integrating the posterior model probabilities for all models that include a given explanatory variable yields the conditional probability that the variable has a nonzero coefficient *p(β ≠ 0)*. To analyze the data we used the ‘bic.glm’ function in the ‘BMA’ package [3] implemented in R (R Development Core Team 2008). In results we report explanatory variables with a posterior probability higher than 50% (Pr(β ≠ 0) > 50).

1 Johnson JB, Omland KS (2004) Model selection in ecology and evolution. Trends Ecol Evol 19: 101-8. doi:S0169-5347(03)00345-8

2 Hoeting JA, Madigan D, Raftery AE, Volinsky CT (1999) Bayesian Model Averaging: A Tutorial. Statistical Science 14: 382-401. doi:10.2307/2676803

3 Raftery AE, Painter IS, Volinsky CT (n.d.) BMA: An R package for Bayesian Model Averaging. R News 5: 2-9
